# RIPK4 function interferes with melanoma cell adhesion and metastasis

**DOI:** 10.1002/1878-0261.70220

**Published:** 2026-02-09

**Authors:** Norbert Wronski, Sławomir Lasota, Ewelina Madej, Anna A. Brożyna, Małgorzata Szczygieł, Agnieszka Harazin‐Lechowska, Jan Czerbniak, Janusz Rys, Jaroslaw Czyz, Agnieszka Wolnicka‐Glubisz

**Affiliations:** ^1^ Department of Biophysics and Cancer Biology, Faculty of Biochemistry, Biophysics and Biotechnology Jagiellonian University Krakow Poland; ^2^ Doctoral School of Exact and Natural Sciences Jagiellonian University Krakow Poland; ^3^ Department of Cell Biology, Faculty of Biochemistry, Biophysics and Biotechnology Jagiellonian University Krakow Poland; ^4^ Department of Human Biology, Institute of Biology, Faculty of Biological and Veterinary Sciences, Institute of Advanced Studies Nicolaus Copernicus University Toruń Poland; ^5^ Department of Tumour Pathology Maria Sklodowska‐Curie National Research Institute of Oncology, Krakow Branch Poland

**Keywords:** amoeboid migration, lung metastasis, melanoma, reprogramming, RIPK4

## Abstract

Receptor‐interacting protein kinase 4 (RIPK4) has been implicated in the progression of numerous tumours. In nonmelanoma skin cancer, RIPK4 plays a suppressor role, whereas in melanoma, it functions as an oncogene that modulates key signalling pathways involved in melanoma cell survival and expansion. Increased RIPK4 levels in metastatic melanoma biopsies prompted us to investigate the consequences of RIPK4 loss for the invasive and metastatic phenotype of melanoma cells. Using an integrated approach involving clinical samples, *in vivo* xenograft models, transcriptomic analysis and 3D functional assays, we show that RIPK4 deletion significantly reduces pulmonary metastasis formation. This reflects its role in late‐stage metastatic events, such as extravasation and colonization, particularly since this phenotype correlates with extensive transcriptional reprogramming of adhesion‐ and motility‐related genes in melanoma cells, as evidenced by next‐generation sequencing and functional validation in spheroid and collagen‐based models. Despite exhibiting features of a partial shift towards an amoeboid phenotype such as membrane blebbing and increased MLC2 phosphorylation, RIPK4 knockout cells display impaired motility and invasion. Re‐expression of RIPK4 restores mesenchymal morphology and migratory capacity. Together, our results establish RIPK4 as a critical regulator of melanoma invasion and metastasis. Nonetheless, they also demonstrate that the loss of RIPK4 function activates compensatory phenotypic shifts in melanoma cells that fail to fully rescue their invasive potential.

## Graphical abstract

RIPK4 promotes melanoma growth and spread. RIPK4 levels increase as skin lesions progress to melanoma. CRISPR/Cas9‐mediated deletion of RIPK4 causes melanoma cells to form less compact spheroids, reduces their migratory and invasive abilities and limits tumour growth and dissemination in mouse models.

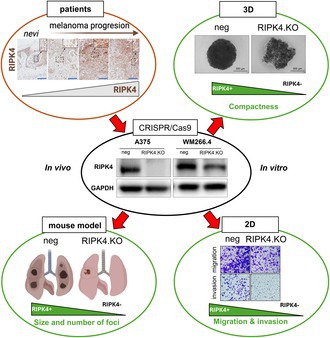

**Abbreviations**
DEG's: differentially expressed genes
ECM: extracellular matrix
EphA2: ephrin type‐A receptor 2
FFPE: formalin‐fixed, paraffin‐embedded
GAPDH: glyceraldehyde‐3‐phosphate dehydrogenase
IMC: integrated modulation contrast
JAM‐C: junctional adhesion molecule C
MLC2: myosin light chain‐2
NOD/SCID: non‐obese diabetic severe combined immunodeficiency
pMLC2: phospho‐myosin light chain‐2
PRAME: preferentially expressed antigen in melanoma
RIPK4: receptor‐interacting protein kinase 4

## 1 Introduction

Malignant melanoma is an aggressive neoplasm originating from melanocytes, which are predominantly localized in the skin. While it primarily affects cutaneous tissue, it may also arise in other melanocyte‐rich sites, such as the uveal tract of the eye (ocular melanoma) and mucosal surfaces [1]. Despite representing only about 1% of all skin cancers, melanoma is responsible for the vast majority of skin cancer‐related deaths due to its resistance to conventional therapies and highly invasive nature. Melanoma cells are characterized by both intrinsic and acquired resistance to cytotoxic agents, driven by impaired drug uptake, enhanced efflux via ATP‐binding cassette (ABC) transporters, evasion of apoptosis and deregulation of signalling pathways such as MAPK and PI3K/AKT [2, 3].

One of the major clinical challenges in melanoma management is its capacity to metastasize. Although early‐stage tumours can often be effectively managed surgically, a substantial proportion of patients eventually develop distant metastases, reducing the overall survival rate to approximately 35% [4]. Recent findings indicate that nearly 50% of patients initially diagnosed with localized melanoma will progress to metastatic disease [5].

Melanoma progression follows a multistep process, beginning with the radial growth phase, during which the tumour expands horizontally through the epidermis. At this stage, the tumour is typically indolent and is considered to lack metastatic potential [6]. The subsequent vertical growth phase marks a critical shift, as melanoma cells infiltrate the dermis, often forming expansile nodules and gaining access to lymphatic and blood vessels, key steps in acquiring the ability to metastasize [7]. These invasive melanomas frequently develop satellite or in‐transit metastases involving regional lymph nodes, while distant metastases including those in the skin, lungs or liver occur in ca. 28% of advanced cases [8].

Among distant organs, lungs represent one of the most common sites of melanoma metastases. This tropism is attributed to the lung's extensive vascular network and its role as a physiological filter for circulating tumour cells, making it particularly susceptible to haematogenous dissemination. The presence of pulmonary metastases is clinically significant, as it correlates with increased tumour burden and poor prognosis [9]. Organ‐specific metastasis is orchestrated by phenotypic reprogramming of melanoma cells and alterations within the tumour microenvironment. This includes changes in chemokine and growth factor secretion, shifts in cellular responsiveness and remodelling of adhesion molecules' expression, particularly of integrins [10].

Receptor‐interacting protein kinase 4 (RIPK4) has emerged as a multifaceted regulator of tumour progression, exhibiting cancer‐type‐specific roles in the regulation of invasion, migration and metastatic spread. Initially recognized for its role in keratinocyte differentiation, RIPK4 is now considered a context‐dependent modulator of multiple signalling pathways implicated in malignancy [11, 12]. In nonmelanoma cutaneous and tongue squamous cell carcinomas, RIPK4 appears to function as a tumour suppressor by inhibiting epithelial‐to‐mesenchymal transition (EMT) and NF‐κB signalling [12, 13]. In contrast, our recent studies in melanoma indicate that RIPK4 displays oncogenic activity, promoting cell migration and invasion via the activation of the NF‐κB and Wnt/β‐catenin pathways [14, 15]. Similarly, in other epithelial‐derived solid tumours such as pancreatic, bladder and ovarian cancers, RIPK4 has been shown to enhance EMT and cellular motility, and its upregulation correlates with poor prognosis [16, 17, 18]. Melanoma invasion relies on EMT‐associated transcriptional programmes and on integrin‐, NF‐κB‐ and Wnt/β‐catenin‐dependent signalling. Notably, these pathways are all reported targets of RIPK4, suggesting that RIPK4 may directly modulate the adhesive and migratory plasticity of melanoma cells.

Despite this, the role of RIPK4 in organ‐specific metastasis, particularly to the lungs, remains largely uncharacterized. To address this gap, we expanded our previous cohort and analysed RIPK4 expression in 155 cutaneous melanoma cases, strengthening the evidence for its association with metastatic progression. In parallel, we employed an *in vivo* model to examine the effects of RIPK4 loss on pulmonary metastasis formation. Furthermore, we characterized RIPK4 activity *in vitro* using both 2D monolayer cultures and 3D melanoma spheroids, which more accurately recapitulate the spatial organization and invasion dynamics of tumour tissue.

## 2 Materials and methods

### 2.1 Clinical samples

RIPK4 expression was analysed in a total of 175 human clinical samples, including formalin‐fixed, paraffin‐embedded (FFPE) tissues from 155 patients diagnosed with cutaneous melanoma and 20 patients with histopathologically confirmed *nevi*. All specimens were archived at the Department of Tumor Pathology, Maria Sklodowska‐Curie National Research Institute of Oncology, Krakow Branch, and collected between 2019 and 2021 as part of routine diagnostic and treatment procedures. Each patient provided written informed consent for the use of redundant tissue obtained during routine diagnostic procedures for research purposes prior to surgery. All tissue samples were anonymized and coded to prevent identification of individual patients. Clinicopathological characteristics of the analysed cohort are summarized in Table 1. The study was conducted in accordance with the ethical principles of the Declaration of Helsinki (1975, revised in 2008) and approved by the Institutional Review Board of Collegium Medicum, Nicolaus Copernicus University (no. KB136/2016) and the Bioethics Committee of Jagiellonian University (no. 1072.6120.125.2017; date of first approval: 28 September 2017, prolonged until 31 December 2025).

**Table 1:** Melanocytic lesions characteristics. Localized melanomas were characterized as melanomas with a Breslow thickness ≤ 1 mm or a Clark level ≤ II.

Variable	Categorization	No. of patients, *n* (%)
*Nevi*	*Nevi*	20 (11.4)
Melanoma site	All	75 (42.9)
	SSM	39 (22.3)
	NM	21 (12.0)
	Other	15 (8.6)
Recurrence		14 (8)
Metastases	All	66 (37.7)
	Lymph nodes	54 (30.9)
	Others	12 (6.9)
Clark	≤ II	32 (18.3)
	III	15 (8.6)
	IV	13 (7.4)
	V	13 (7.4)
	Lack of data	2 (1.1)
Breslow	≤ 1 mm	26 (15.4)
	1.1–2 mm	5 (2.9)
	2.1–3 mm	6 (3.4)
	3.1–4 mm	6 (3.4)
	> 4 mm	26 (14.9)
	Lack of data	5 (2.9)

### 2.2 Immunohistochemistry for RIPK4 and section assessment

RIPK4 expression in tissue samples was detected using immunohistochemistry, as previously described [14]. Sections were evaluated under the BX41 microscope (Olympus Optical Co., Tokyo, Japan), and the image documentation was prepared using the ColorView III camera (Soft Imaging System, Hanover, Germany) and analysis 3.2 software (Soft Imaging System, Hanover, Germany). Semiquantitative scoring of RIPK4 staining was performed separately for heterogeneous, uniform and granular staining patterns according to previously established criteria [14].

### 2.3 Cell culture

Human melanoma cell line A375 (RRID: CVCL_0132) was obtained from ATCC (Manassas, VA, USA) in 2020, and WM266.4 (RRID: CVCL_2765) was kindly provided by the Department of Medical Biochemistry, Jagiellonian University Medical College (Kraków, Poland) in 2006. The identity of both cell lines was verified by STR profiling at the Genomics Core Facility, Jagiellonian University, in 2025, ensuring authentication within the past 3 years. STR profiling was performed by analysing 14 standard loci including the Amelogenin locus, and the resulting profiles were compared with reference profiles to confirm cell line identity. Phenotypically stable sublines of human melanoma cell lines with varying degrees of RIPK4 downregulation (A375^RIPK4.KO^ and WM266.4^RIPK4.KO^) and their RIPK4.KO‐negative (neg) counterparts were previously generated using the CRISPR/Cas9 system [15]. Gene targeting was performed using a lentiviral sgRNA construct specific to RIPK4 (ID: CRISPR1029813_LV; Invitrogen™, Thermo Fisher Scientific, Waltham, MA, USA), while negative controls were generated using a nontargeting sgRNA with no homology to the human genome (Cat. No. A32063; Invitrogen™, Thermo Fisher Scientific), as previously described [15] (see Fig. S1). Cells were cultured in RPMI‐1640 medium supplemented with 10% FBS (Gibco, Thermo Fisher Scientific, Waltham, MA, USA) and antibiotics (penicillin 150 U·mL^−1^, streptomycin 100 μg·mL^−1^), at 37 °C in a humidified atmosphere of 5% CO_2_. All experiments were performed using cells confirmed to be free of mycoplasma contamination, which was routinely tested every 6 months.

### 2.4 Xenografts experimental metastatic assays

Forty‐four female NOD/SCID (non‐obese diabetic severe combined immunodeficiency) mice, aged 6–8 weeks, were purchased from Janvier Labs (Le Genest‐Saint‐Isle, France). Animals were maintained under specific pathogen‐free (SPF) conditions with *ad libitum* access to food and water. RIPK4.KO‐negative (neg) and RIPK4 knockout (RIPK4.KO) sublines of A375 (0.5 or 1 × 10^6^ cells) or WM266.4 (1.5 × 10^6^ cells) were separately suspended in 100 μL of ice‐cold PBS and intravenously injected into the tail veins of the mice (*n* = 9–13 per group). After 6 weeks, animals were euthanized via intraperitoneal injection of a xylazine/ketamine overdose. Lung tissue samples were resected and fixed according to a standard FFPE protocol. Tissue sections were stained with haematoxylin and eosin (H&E) for evaluation of tumour burden and progression. Ten tissue sections were obtained from each lung, with the third and seventh sections stained using haematoxylin and eosin (H&E). The entire cross section of each section was examined, and all slides were digitally scanned. The number of foci was quantified manually after pathological evaluation. The foci area was normalized to 100% of the total lung area using the imagej software (National Institutes of Health, Bethesda, MD, USA). The number and the percentage of the affected surface area were assessed in a blinded manner. Following the assessment, all data were matched to their corresponding sample identifiers.

RIPK4 expression was detected immunohistochemically, as previously described [14, 19]. Staining intensity (SI) was assessed using a 4‐point scale, from 0 to 3, with 0 as negative (0), weak (1), moderate (2) and strong (3) in relation to the positive control (duodenum). In addition, the percentage of immunoreactive cells was also assessed (IR). These data were used for calculation of a semiquantitative score as follows: SQ = IR × SI. Staining intensity and percentage of immunoreactive cells were evaluated manually in a blinded manner after assessment; the data were paired with sample number. The whole section was evaluated.

Preferentially Expressed Antigen in Melanoma (PRAME) was detected immunohistochemically, as it is routinely used to distinguish *nevi* from melanoma, including dysplastic *nevi* from nevus‐associated melanoma *in situ* [20, 21, 22]. FFPE sections (4 μm) were deparaffinized, rehydrated and subjected to antigen retrieval (64 min at 100 °C; using ULTRA CC1 buffer; Ventana (Ventana Medical Systems, Inc.), Roche (Tucson, AZ, USA)) followed by endogenous peroxidase quenching (Peroxidase Inhibitor; Ventana, Roche). Sections were incubated with a primary rabbit monoclonal anti‐PRAME antibody (EPR20330; Ventana, Roche) for 32 min at 36 °C. Immunoreactivity was visualized using the OptiView DAB IHC Detection Kit (Ventana, Roche). Sections were additionally counterstained with haematoxylin. All experiments involving animals were approved by the II Local Ethics Committee of the Institute of Pharmacology of the Polish Academy of Sciences (approval numbers 82/2023, 135/2023 and 52/2024; dates: 23 April 2023 25 January 2024). All procedures were conducted in accordance with the Committee's ethical guidelines.

### 2.5 Migration and invasion assay

Qualitative and quantitative analysis of migration and invasion was carried out as described by Wronski et al. [15]. Briefly, melanoma cells (1 × 10^5^/well) were seeded into the upper chambers of Transwell inserts (8 μm pores; Merck Millipore, Burlington, MA, USA). Migration was assessed after 20 h, and invasion after 48 h using Geltrex‐coated filters (Thermo Fisher Scientific). RPMI‐1640 with 10% FBS served as a chemoattractant. After incubation, cells were fixed, stained with 0.2% crystal violet (Merck Millipore), and analysed microscopically (Olympus, Tokyo, Japan). Quantification was performed using CellTracker Red fluorescence (λ_ex_ 577 nm, λ_em_ 602 nm) based on standard curves for each cell line.

### 2.6 Single‐cell migration analysis and cytoskeleton visualization in a 2D system

#### 2.6.1 Time‐lapse imaging and migration analysis

Migration analysis was conducted using time‐lapse microscopy. Cells were seeded 24 h before the experiment at a density of 2 × 10^4^ cells per well of a 12‐well plate. Imaging was performed on a fully motorized Leica DMI 6000B microscope (Leica Microsystems, Wetzlar, Germany) equipped with an environmental chamber and CO_2_ controller (Pecon, Erbach, Germany), using an HC PL FLUOTAR 10×/0.30 DRY objective, integrated modulation contrast (IMC), and a Leica DFC360 FX camera controlled by Leica Application Suite X (las x) 3.4 software (all Leica Microsystems). Time‐lapse images were captured for multiple fields of view every 10 min over an 18‐h period. Image series were analysed with the hiro 1.0.0.4 software (W. Czapla, Kraków, Poland), following previously described methods [23]. Cell centroids were manually tracked to construct migration trajectories, which were brought to an origin of coordinate system to generate circular diagrams. Migration speed (trajectory length/recording time) and displacement (distance between trajectory start and end) were further calculated from cell trajectories.

#### 2.6.2 Immunofluorescence and imaging of cytoskeleton

Cells were seeded at a density of 2 × 10^4^ cells per well in a 12‐well plate containing sterile glass coverslips. After 24 h, the cells were fixed with 3.7% formaldehyde for 15 min, permeabilized using 0.1% Triton X‐100 for 5 min and blocked with 3% BSA (bovine serum albumin) for 60 min. For staining, cells were incubated overnight with mouse antivinculin antibody (V9131; 1 : 400; Sigma‐Aldrich, St. Louis, MO, USA), followed by a 2‐h incubation with goat anti‐mouse AF488‐antibody (A11029; 1 : 300), AF568‐phalloidin (A12380; 1 : 40) and Hoechst 33258 (H1398; 2 μg·mL^−1^; all Thermo Fisher Scientific), after which aqueous specimens were prepared. The samples were imaged using a Leica DMI 6000B microscope equipped with a TIRFM module, an HC PL APO 100×/1.47 OIL objective, a DFC360FX camera, a metal halide illuminator and a laser unit operating at 488 nm (all Leica Microsystems). The evanescent wave penetration depth in the TIRF channel was set to 110 nm. Images were captured in epifluorescence (actin and nuclei), TIRFM (vinculin) and DIC (differential interference contrast). The las x 3.4 software was used for equipment control and subsequent image processing.

### 2.7 Single‐cell migration analysis and cytoskeleton visualization in a 3D system

#### 2.7.1 Preparation of collagen gel

The collagen matrix (1.5 mg·mL^−1^) was prepared using bovine collagen I (A1064401; Thermo Fisher Scientific) as previously described [24], following the manufacturer's protocol with slight modifications. Glass‐bottom chambers (μ‐Slide 8 Well high Glass Bottom; ibidi GmbH, Gräfelfing, Germany) were used. Initially, a cell‐free collagen gel layer was applied (90 μL per well), followed by an application of a second layer containing evenly distributed cells (8 × 10^4^ per well, suspended in 200 μL of gel). The gels were prepared using RPMI‐1640 medium (5× concentrated, prepared from powder; Sigma‐Aldrich) and supplemented with FBS to a final concentration of 10%. After polymerization, the wells were filled with 300 μL of complete culture medium, which was optionally supplemented with 5 μm blebbistatin or 10 μm Y27632 (inhibitors of myosin II and ROCK1/2, respectively; both Sigma‐Aldrich).

#### 2.7.2 Time‐lapse imaging

Time‐lapse imaging was performed 1 h after cells were seeded using a microscope described above (Section 2.6.1). Z‐stacks with a total thickness of approximately 1 mm and a step size of 8 μm were acquired with HC PL FLUOTAR L 20×/0.40 DRY objective every 5 min over 3–6 h using IMC. The obtained image series were subjected to maximum projection within two equal regions per stack (positioned at different levels) and used for single‐cell migration analysis, performed as described previously (Section 2.6.1).

#### 2.7.3 Actin cytoskeleton staining

Following the 3‐h imaging session, cells were fixed with 3.7% formaldehyde for 20 min, permeabilized with 0.5% Triton X‐100 for 10 min and blocked with 3% BSA for 60 min. Cells were then incubated with AF568‐phalloidin (A12380; 1 : 20) and Hoechst 33258 (H1398; 5 μg·mL^−1^; both Thermo Fisher Scientific).

#### 2.7.4 Fluorescence imaging

Prepared samples were imaged using a Leica DMi8 microscope equipped with a pE‐4000 LED illuminator (CoolLED Ltd., Andover, UK), set at 365 and 550 nm, QUAD‐S filter set, a Leica DFC7000GT camera, all under control of las x 3.7 software (all Leica Microsystems). Z‐stacks were acquired with N PLAN L 20×/0.35 DRY and HC PL APO 40×/1.30 OIL objectives using step sizes of 3.16 μm (covering approximately 700 μm) or 0.54 μm (covering 70–100 μm), respectively. The acquired stacks were subjected to blind deconvolution and visualized in maximum projection mode using the las x 3.7 software.

### 2.8 Western blot

Western blot analysis was performed as described previously [25]. Membranes were cut prior to hybridization with the following human‐specific primary antibodies: RIPK4 (cat. no. 12636), GAPDH (cat. no. 5147), EphA2 (cat. no. 6997), MLC2 (cat. no. 8505), pMLC2 (cat. no. 3674), MCAM (cat. no. 81701), N‐cadherin (cat. no. 4061), Integrin αV (cat. no. 4711), Thrombospondin‐1 (cat. no. 7879), Sox‐9 (cat. no. 82630) (all from Cell Signaling Technology, Danvers, MA, USA), JAM‐C (cat. no. A303‐761A; Bethyl Laboratories, Thermo Fisher Scientific, Waltham, MA, USA), ITGA2 (cat. no. 30703‐1‐AP; Proteintech Group, Rosemont, IL, USA). For detection of proteins with similar molecular weights, membranes were stripped and subsequently re‐incubated with the appropriate primary antibodies. The secondary antibody used was an HRP‐conjugated goat anti‐rabbit IgG (cat. no. 7074; Cell Signaling Technology). Signal detection was performed using Clarity Western ECL Substrate (Bio‐Rad, Laboratories, Hercules, CA, USA) and visualized with the ChemiDoc imaging system (Bio‐Rad). Band intensities were quantified using the imagelab 5.2.1 software (Bio‐Rad). Densitometric quantification of the analysed protein levels was normalized to the corresponding GAPDH band obtained from the same membrane. Representative GAPDH is shown in the figures.

### 2.9 RNA isolation and sequencing (RNA‐seq)/next‐generation sequencing analysis

Total RNA sequencing (RNA‐seq) was performed on A375^neg^ and A375^RIPK4.KO^ cells. Total RNA was isolated using Total RNA Mini Plus (A&A Biotechnology, Gdansk, Poland) according to the manufacturer's recommendations. RNA concentration and purity were determined with CLARIOstar Plus microplate reader (absorbance at 260/280 nm and 260/230 nm; BMG LABTECH, Ortenberg, Germany). Isolates were subjected to poly(A) enrichment using NEBNext Poly(A) mRNA Magnetic Isolation Module (New England Biolabs, Ipswich, MA, USA). Strand‐specific cDNA libraries were constructed with NEBNext Ultra™ II Directional RNA Library Prep Kit for Illumina® (New England Biolabs). The library preparation, RNA‐seq, and RNA‐seq data preparation were performed by Genomed (http://www.genomed.pl/, Warszawa, Poland). The library size was checked with the Bioanalyzer 2100 High‐Sensitivity (Agilent Technologies, Santa Clara, CA, USA) chip and quantified using the qPCR method. Sequencing was performed on NovaSeq 6000 (Illumina, San Diego, CA, USA) in PE150 mode.

### 2.10 Spheroid formation and analysis

Three‐dimensional spheroids were generated from 6 × 10^3^ melanoma cells (RIPK4.KO and their respective negative controls) as described previously [26]. Cell suspensions for 20 spheroids were prepared by suspending 1.2 × 10^5^ cells in 75 μL of 2.8% methylcellulose (R&D System, Minneapolis, MN, USA) in complete culture medium, followed by adjustment to a final volume of 600 μL. To promote spheroid formation, 30 μL droplets (6 × 10^3^ cells per droplet) were dispensed onto the inner surface of 60‐mm Petri dish lids. The dish bottoms were filled with 5 mL of PBS to maintain humidity. Lids were then inverted and placed over the PBS‐containing dishes and incubated at 37 °C for 4 days. Images of spheroids were acquired using an Olympus IX73 microscope (Olympus) and a DLT‐Cam PRO 5MP camera (Delta Optical, Minsk Mazowiecki, Poland) at 10× magnification. Spheroid area was measured from captured images using imagej software (National Institutes of Health). Twenty spheroids per group were analysed, and data were collected from four independent experiments.

### 2.11 RNA isolation for TaqMan array human extracellular matrix and adhesion molecules array

Selected gene expression in spheroids was assessed by real‐time qPCR. Total RNA was isolated from six pooled spheroids per sample using the TaqMan™ Fast Advanced Cells‐to‐Ct Kit (Invitrogen™, Thermo Fisher Scientific). Cell lysates were reverse transcribed using the RT Enzyme Mix provided in the kit, in a final reaction volume of 50 μL. Next, the cDNA was diluted with nuclease‐free water to a final volume of 150 μL. Each qPCR reaction consisted of 10 μL of diluted cDNA, 10 μL of RT HS‐PCR Mix (A&A Biotechnology), and 1 μL of TaqMan probe (cat. no. 4414133; Thermo Fisher Scientific). Amplification was performed in a 96‐well optical plate using a qTOWER^3^ thermal cycler (Analytik Jena, Jena, Germany). The relative gene expression levels were calculated using the $2^{-\Delta \Delta C_{t}}$ method, with GAPDH as the reference gene.

### 2.12 Re‐expression of RIPK4 in knockout cells

To reintroduce RIPK4 expression, A375^RIPK4.KO^ cells were transfected with a RIPK4‐expressing plasmid, pRP(Exp)_EGFP/Neo‐CMV>hRIPK4/FLAG, or an empty vector control, pRP(Exp)‐CMV>EGFP (VectorBuilder Inc., Chicago, IL, USA) using Lipofectamine™ 2000 (cat. no. 11668019; Thermo Fisher Scientific), according to the manufacturer's instructions. Briefly, 2 × 10^5^ cells were seeded in 35‐mm culture dishes 24 h prior to transfection. For the transfection procedure, 7 μg of plasmid DNA was diluted in 150 μL of Opti‐MEM™ medium (cat. no. 31985070; Thermo Fisher Scientific), and separately, 7 μL of Lipofectamine 2000 was mixed with 150 μL of Opti‐MEM. Both mixtures were incubated for 15 min at room temperature, then combined and incubated for an additional 30 min to allow complex formation. Then, the prepared mixture was added dropwise to the cells in 700 μL of Opti‐MEM and incubated for 24 h under standard culture conditions. Subsequently, the medium was replaced with fresh complete culture medium, and the cells were cultured for an additional 24 h. Successful transfection and RIPK4 expression were verified by assessing GFP fluorescence and western blot analysis.

### 2.13 Statistics

Data are presented as mean ± SD, unless otherwise indicated in the figure legends. Statistical tests used are indicated in figure legends. Statistical analyses were performed using graphpad prism software (version 9.0; GraphPad Software, La Jolla, CA, USA). Differences between groups were assessed using one‐way ANOVA or two‐tailed unpaired Student's *t*‐test, as appropriate. Statistical significance was defined as *P* < 0.05 (*), *P* < 0.01 (**), *P* < 0.001 (***), and *P* < 0.0001 (****), consistent with the notations used in the figures.

## 3 Results

### 3.1 RIPK4 is upregulated in metastatic melanoma compared to nevi

**Fig. 1:**
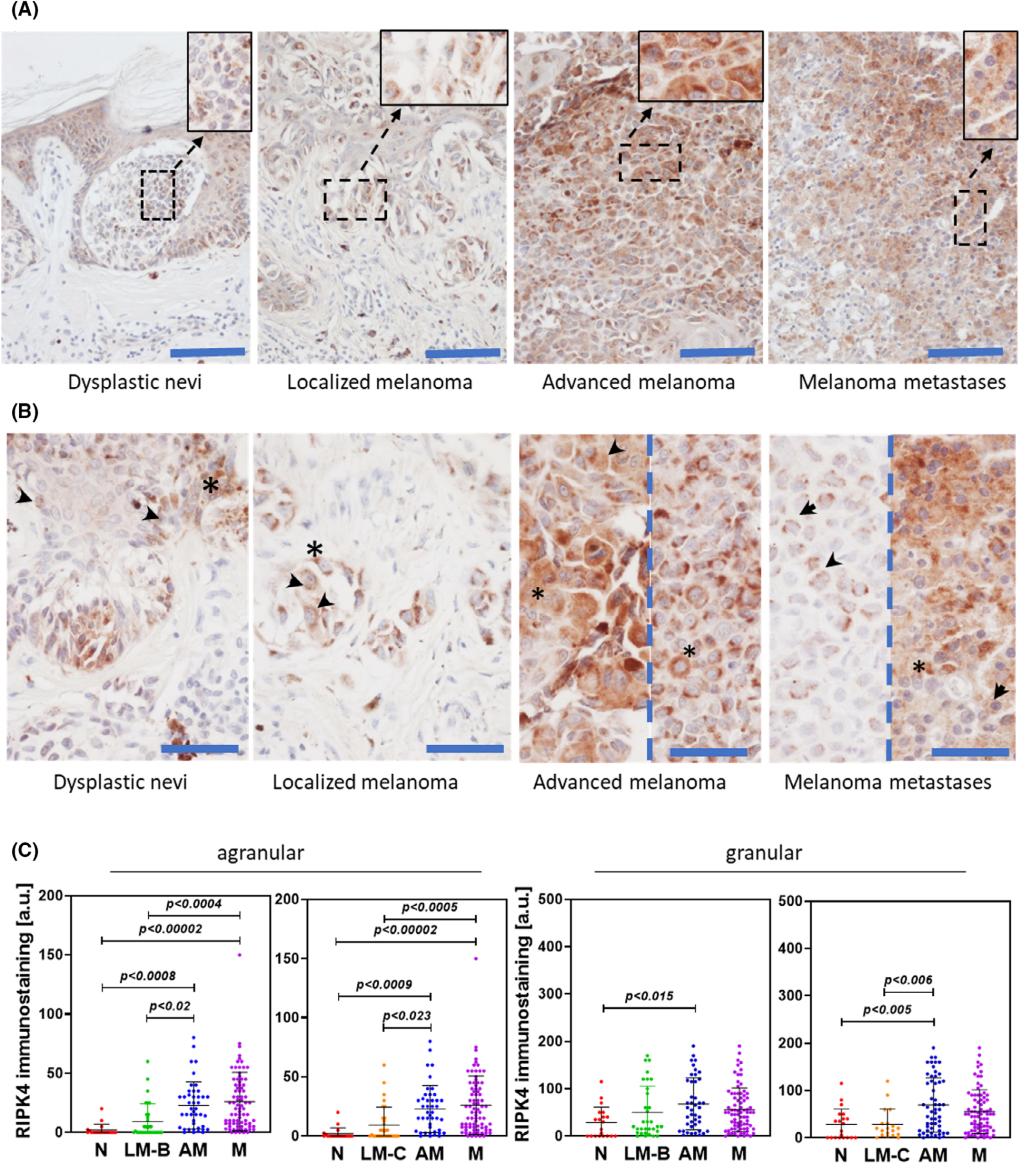
Stage‐dependent RIPK4 expression in clinical samples. (A) Representative images of RIPK4 immunostaining in human *nevi*, primary localize and advanced cutaneous melanomas and metastatic melanomas. Boxed regions are shown at higher magnification in the inset. Scale bar = 100 μm. (B) Higher‐magnification images of RIPK4 immunostaining in human *nevi*, primary cutaneous, and metastatic melanomas. Scale bar = 50 μm. Dotted lines separate images from two different melanoma cases. Arrows indicate granular RIPK4 staining, asterisks indicate agranular staining. (C) Densitometric quantification of RIPK4‐specific agranular and granular staining in tissue specimens. Graphs show the mean ± SD for dysplastic *nevi* (N, *n* = 20 patients); localized melanoma Breslow ≤ 1 mm (LM‐B, *n* = 32 patients); localized melanoma Clark ≤ II (LM‐C, *n* = 32 patients); advanced melanoma (AM; *n* = 57 patients); metastases (M; *n* = 66 patients). All samples were evaluated in a blinded manner. Statistical analysis was performed using ANOVA.

Our previous studies, based on seven clinical samples, showed the presence of RIPK4 in melanomas and revealed a distinct staining pattern (agranular and granular) was observed. Preliminary data also indicated a potential role for RIPK4 in advanced‐stage melanoma [14]. Here, we expanded this analysis by including a larger cohort of 175 patients with melanocytic lesions (Table 1). Among these, 11.4% were classified as *nevi*, while 42.9% and 37.8% represented primary and metastatic melanomas, respectively; the remaining 8% were derived from recurrent cases. RIPK4 was detected in the cytoplasm of tumour cells in two distinct patterns—agranular (indicated by arrows) and granular (indicated by asterisks), as we had previously reported [14, 19]. Each pattern exhibited a different intensity of staining (Fig. 1A,B). RIPK4 levels were significantly lower in melanocytes in *nevi* and dermal fibroblasts compared to keratinocytes and melanoma cells in both staining patterns, with stronger statistical differences observed in the agranular pattern. In this pattern, statistically higher staining intensity was observed in advanced melanomas and melanoma metastases than in *nevi* and localized melanomas. For the granular pattern, statistically significant differences were found only between *nevi* and advanced melanomas (*P* < 0.005), and between localized and advanced melanomas (*P* < 0.005), when grouped according to Clark's level. Moreover, RIPK4 expression positively correlated with disease stage, particularly in the agranular staining pattern, where the differences between groups were statistically significant (Fig. 1C). These findings support a model in which RIPK4 contributes to melanoma progression.

### 3.2 Loss of RIPK4 expression impairs pulmonary melanoma metastasis

**Fig. 2:**
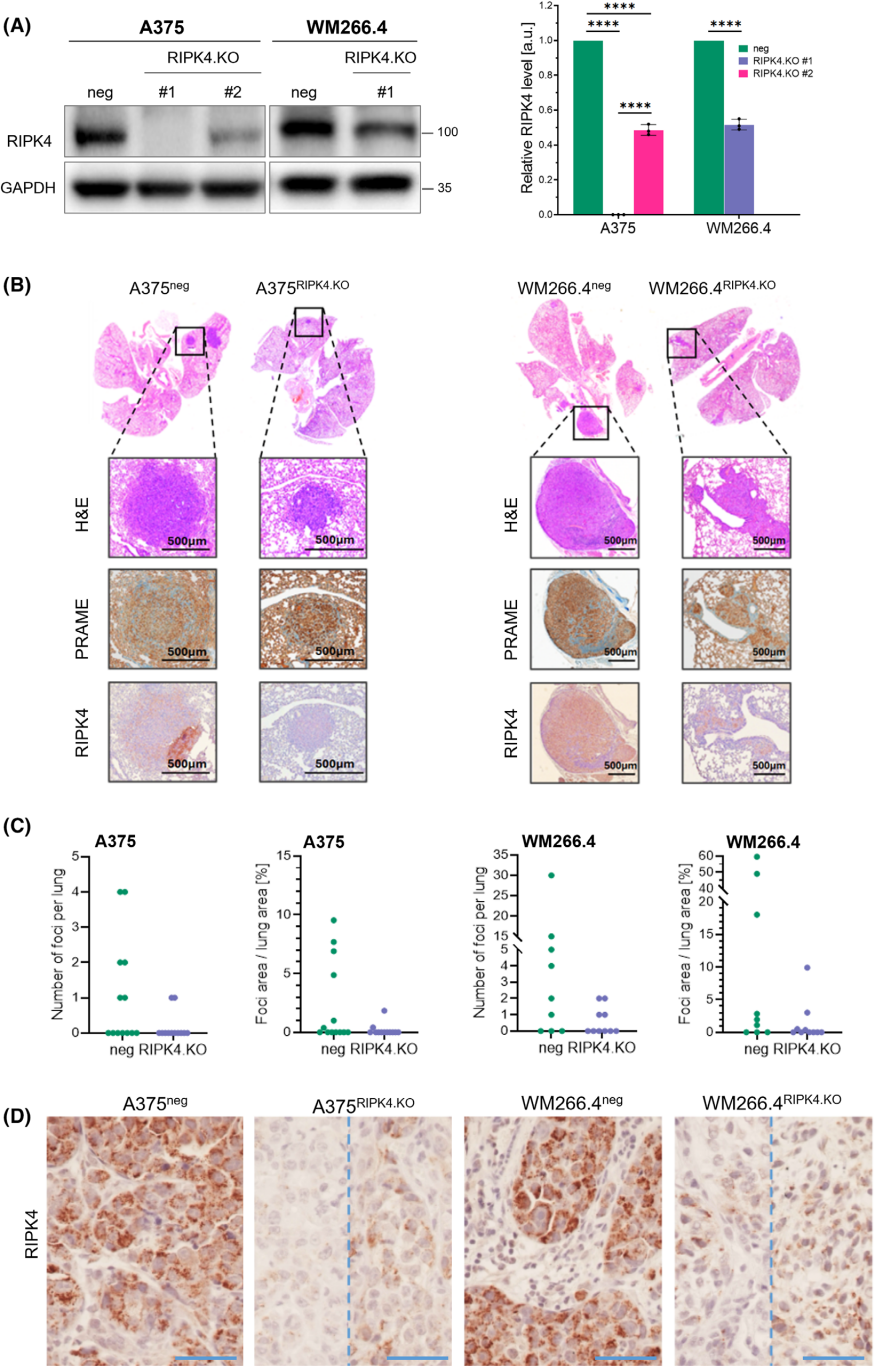
RIPK4 downregulation impairs the lung colonization capacity of melanoma cells. (A) Western blot analysis of RIPK4 protein levels in A375^RIPK4.KO^ (clones #1 and #2) and WM266.4^RIPK4.KO^ cells, together with their respective negative controls, including densitometric quantification. GAPDH served as a loading control. Data are presented as mean ± SD from three independent biological replicates (*n* = 3). (B) Representative H&E, PRAME, and RIPK4 immunohistochemical staining of lung sections obtained from NOD/SCID mice injected intravenously with A375^neg^ (*n* = 13), A375^RIPK4.KO^ (clone #1; *n* = 12), WM266.4^neg^ (*n* = 9), and WM266.4^RIPK4.KO^ (*n* = 10) cells. Scale bar = 500 μm. (C) Dot plot representing the number and total area of metastatic foci in lung sections. Animals were randomly assigned to experimental groups, and histological analysis was performed blinded. (D) Higher‐magnification immunohistochemical staining of RIPK4 in melanoma lesions within the lung, derived from xenografts of RIPK4.KO and corresponding negative‐control cells. Dotted lines separate images from two different melanoma lesions. Scale bar = 50 μm. Statistical analysis was performed using ANOVA or two‐tailed unpaired Student's *t*‐test, ****P < 0.0001.

Loss of RIPK4 function in A375^RIPK4.KO^ and WM266.4^RIPK4.KO^ cells was previously shown to significantly reduce tumour growth in SCID mice [15]. To determine whether RIPK4 is also involved in melanoma dissemination to distant organs, we focussed on pulmonary metastasis formation. NOD/SCID mice were intravenously injected via the tail vein with either RIPK4.KO or negative control variants of A375 and WM266.4 melanoma cells (Fig. 2A–D). Western blot analysis confirmed complete RIPK4 loss in A375^RIPK4.KO^ clone #1 and ~ 50% reduction in clone #2 and WM266.4^RIPK4.KO^ cells (Fig. 2A). This approach allows for the assessment of late‐stage metastatic events, including extravasation and colonization of distant organs by circulating melanoma cells. Given melanoma's strong tropism for the lungs [27], we quantified the presence, number, and size of human melanoma foci in the lungs of NOD/SCID mice (Fig. 2B–D). Tumour detection was based on a combination of H&E and PRAME staining. As shown in Fig. 2B, lung metastases were observed in only 2 out of 12 (17%) mice injected with A375^RIPK4.KO^ cells and in 4 out of 10 (40%) mice injected with WM266.4^RIPK4.KO^ cells. In contrast, the control groups exhibited higher metastatic incidence: six out of 13 (46%) mice for A375 and six out of nine (67%) for WM266.4. Concomitantly, the foci formed by RIPK4.KO variants of both cell lines were less abundant and smaller than those formed by negative control cells. Immunohistochemical staining confirmed the absence or markedly reduced expression of RIPK4 in pulmonary lesions formed by KO variants (Fig. 2D). These data confirm the importance of RIPK4 for melanoma invasion and demonstrate its involvement in the late metastatic colonization of this cancer.

### 3.3 RIPK4 loss impairs melanoma cell motility via altered adhesion molecule profiles

**Fig. 3:**
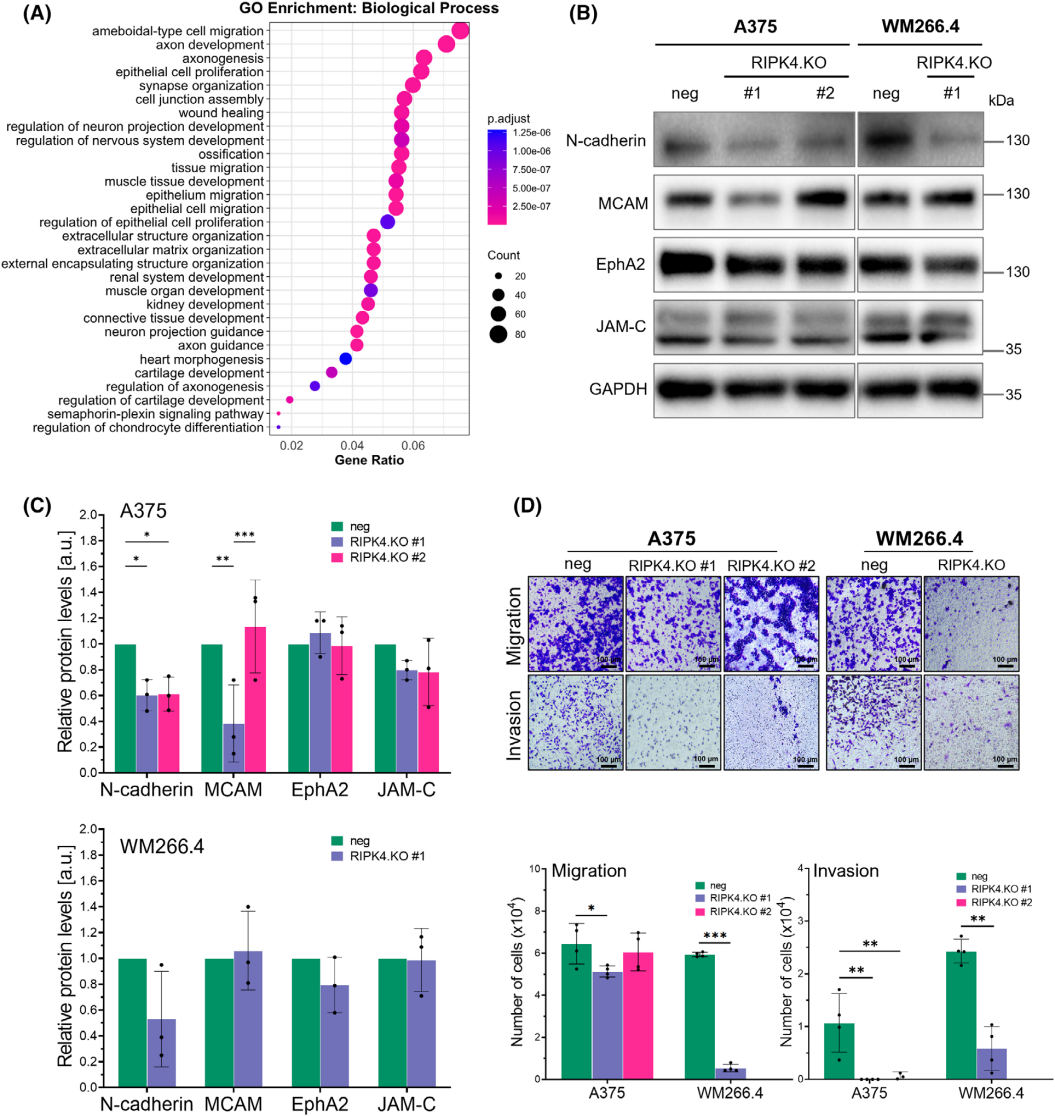
RIPK4 regulates melanoma cell motility via adhesion molecules. (A) GO enrichment analysis of differentially expressed genes (DEGs) identified by RNA‐seq following RIPK4 knockout in A375 cells. The bubble plot displays the top 30 significantly enriched biological processes. The size of each bubble represents the number of DEGs associated with each term, and the colour indicates statistical significance. (B) Western blot analysis of N‐cadherin, MCAM, EphA2 and JAM‐C expression levels in RIPK4.KO and control cells. GAPDH served as a loading control. A representative GAPDH band is shown. (C) Densitometric quantification of analysed protein levels, normalized to the corresponding GAPDH band obtained from the same membrane and the same sample. Data are presented as mean ± SD from three independent biological replicates (*n* = 3). (D) Analysis of A375 and WM266.4 cells with RIPK4 knockout (RIPK4.KO) and their respective negative controls. Transmigration (top) and invasion (bottom) assays. Scale bar = 100 μm. Bar plots show quantification of cell migration and invasion, presented as mean ± SD from three independent experiments (*n* = 3). Statistical analysis was performed using ANOVA or two‐tailed unpaired Student's *t*‐test, *P < 0.05, **P < 0.01, ***P < 0.001.

To explore the molecular mechanisms through which RIPK4 contributes to metastasis, we performed RNA‐seq on A375^RIPK4.KO^ and control cells. PCA, expression density, and volcano plot analyses (Fig. S2a–c) demonstrated clear separation between experimental groups, supporting robust transcriptomic differences. Consistently, the analysis revealed statistically significant alterations in 726 protein‐coding transcripts that were either up‐ or downregulated by at least 50% (*P* < 0.05). Gene Ontology enrichment indicated differential expression of genes associated with ‘amoeboidal‐type cell migration’, ‘epithelial cell migration’ and ‘extracellular matrix organization’ (Fig. 3A, see Fig. S2d,e). These findings highlight a crucial role for RIPK4 in regulating cell motility as well as the adhesive and invasive phenotype of melanoma cells.

Cancer cell extravasation and colonization of distant organs critically depend on the adhesive properties of circulating tumour cells. Therefore, we further analysed the expression of adhesion molecules previously implicated in melanoma lung metastasis [28, 29, 30]. In two‐dimensional monolayer cultures, RIPK4 knockout resulted in a modest reduction of approximately 20% in JAM‐C expression in both A375^RIPK4.KO^ clones, while no changes were observed in the WM266.4 line (Fig. 3B,C). Interestingly, a statistically significant downregulation of MCAM expression was observed in A375^RIPK4.KO^ clone #1 (~ 60%; *P* < 0.01), whereas clone #2 exhibited a slight, non‐significant increase (~ 20%) and no change was detected in WM266.4^RIPK4.KO^ cells. Although the results were not consistent across both clones, these findings suggest that MCAM expression may be variably affected by RIPK4 knockout. In contrast, N‐cadherin expression was consistently reduced by approximately 50% in RIPK4.KO cells of both melanoma lines (*P* < 0.05 for both A375^RIPK4.KO^ clones). Meanwhile, EphA2 expression remained unaffected by RIPK4 knockout in either A375 or WM266.4 cells.

Given the *in vivo* evidence indicating reduced metastatic potential following RIPK4 knockout, we also assessed whether RIPK4 directly regulates melanoma cell migration and invasion *in vitro*. Transwell‐based assays revealed a substantial reduction in both migratory and invasive capacities of RIPK4.KO cells compared to their respective negative controls (Fig. 3D). Notably, in the A375 cell line, loss of RIPK4 function resulted in a modest reduction in migration across uncoated membranes compared to control cells, with clone #1 exhibiting a 20% decrease (*P* < 0.05) and clone #2 a nonsignificant 6% reduction (*P* = 0.76). In contrast, invasion through Geltrex‐coated membranes was almost completely abolished in both clones (~ 100% reduction; *P* < 0.01). The WM266.4 cell line showed a consistent reduction of around 91% in migration (*P* < 0.001) and 76% in invasion (*P* < 0.01) upon RIPK4 knockout. Interestingly, this inhibitory effect was observed despite incomplete RIPK4 knockout in WM266.4^RIPK4.KO^ cells, suggesting a high sensitivity of this line to RIPK4‐dependent regulation of invasiveness.

### 3.4 RIPK4 knockout disturbs spheroid cohesion and extracellular matrix organization (ECM)

**Fig. 4:**
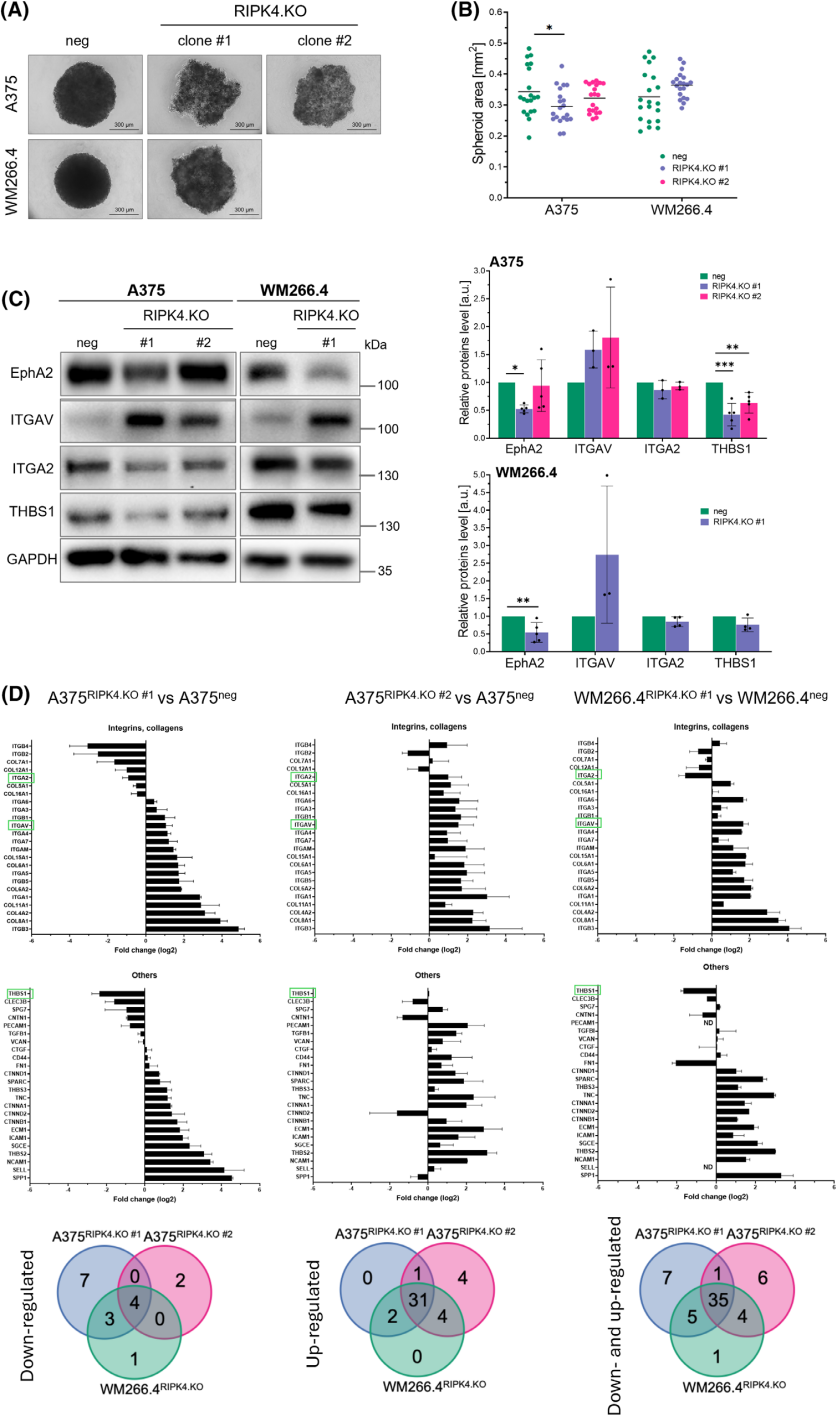
RIPK4 interferes with the adhesive status of melanoma cells in 3D spheroid model. (A) Phase‐contrast images of spheroids formed by RIPK4.KO and negative‐control cells. Scale bar = 500 μm. (B) Quantification of spheroid area. Each dot represents an individual spheroid (20 spheroids per condition). Data are shown as mean ± SD from four independent biological experiments (*n* = 4), each performed in quintuplicate. (C) Western blot analysis of EphA2, ITGAV, ITGA2 and THBS1 protein levels in RIPK4.KO and control spheroids. GAPDH was used as a loading control. Data are presented as mean ± SD from three independent biological experiments for integrin αV and integrin α2 (*n* = 3‐4) and five independent biological experiments for EphA2 and THBS1 (*n* = 4‐5), with 10 spheroids pooled per sample. (D) Relative mRNA expression levels of ECM‐related genes in spheroids assessed using the TaqMan Array Human Extracellular Matrix and Adhesion Molecules panel. Data are presented as fold change relative to the negative control from two independent biological experiments (*n* = 2), with six spheroids pooled per sample. These data are also visualized using Venn diagrams showing genes uniquely upregulated, uniquely downregulated, or shared between the groups. Circles represent A375^RIPK4.KO^ clone #1 (blue), clone #2 (pink), and WM266.4^RIPK4.KO^ (green) cells. Statistical analysis was performed using ANOVA or two‐tailed unpaired Student's *t*‐test *P < 0.05, **P < 0.01, ***P < 0.001.

To better reflect the spatial architecture and cell–cell interactions of melanoma cells, we next investigated the consequences of RIPK4 knockout using 3D spheroid models. In this system, RIPK4 knockout significantly impaired spheroid formation. As shown in Fig. 4A,B, both negative controls (A375^neg^ and WM266.4^neg^) and A375^RIPK4.KO^clone #2 cells effectively formed compact, spherical architecture with smooth contours, typical of invasive melanoma aggregates. In contrast, spheroids formed by A375^RIPK4.KO^ clone #1 and WM266.4^RIPK4.KO^ cells appeared less cohesive, exhibiting irregular morphology and disrupted borders, suggesting impaired cell–cell adhesion. Moreover, spheroid size was markedly reduced in A375^RIPK4.KO^ clone #1 compared to the control (by 13.5%; *P* < 0.05), while clone #2 displayed an intermediate phenotype. These morphological changes highlight the impact of RIPK4 loss on spheroid structural integrity. Surprisingly, we observed significantly reduced EphA2 expression in A375^RIPK4.KO^ clone #1 and WM266.4^RIPK4.KO^ spheroids (Fig. 4C), suggesting that its regulation may be dependent on cell–cell contact or the multicellular organization within the 3D architecture. Interestingly, despite these adhesive deficits, both cell lines exhibited increased expression of integrin αV (ITGAV), while integrin α2 (ITGA2) slightly decreased, possibly reflecting a compensatory mechanism aimed at preserving residual adhesive function in RIPK4‐deficient spheroids. In addition, both cell lines showed a reduction in thrombospondin 1 (THBS1) expression (24% in WM266.4 and 58% (clone #1) and 37% (clone #2) in A375), an integrin‐mediated adhesion protein that further supports the notion of globally weakened adhesive signalling in RIPK4‐deficient cells (Fig. 4C). Notably, protein‐level analyses performed in 2D cultures confirmed these trends, revealing an even stronger decrease in both ITGA2 and THBS1 (see Fig. S3).

To explore the molecular underpinnings of RIPK4‐dependent alterations in spheroid architecture, we performed mRNA profiling of 50 ECM‐related genes, including those involved in connective tissue organization, cell adhesion, transmembrane inhibition, basement membrane components and collagen scaffolding. As shown in Fig. 4D, the expression of several ECM‐associated genes was markedly dysregulated upon RIPK4 knockout in both A375 and WM266.4 cell lines. In A375^RIPK4.KO^ clone #1, we observed upregulation of 17 genes encoding collagens and integrins, including *COL1A1*, *COL1A2*, *COL3A1* and *COL6A3*, all showing fold changes exceeding log_2_FC > 2. Several adhesion receptors such as *ITGB3*, *ITGA2* and *ITGA5*, which are central to ECM binding and mechanotransduction, were also significantly increased. A similar pattern was observed in A375^RIPK4.KO^ clone #2 and WM266.4^RIPK4.KO^ cells, although transcript‐level changes were generally less pronounced in some transcripts, suggesting clonal heterogeneity in RIPK4‐mediated transcriptional regulation. Interestingly, while six genes were downregulated in A375^RIPK4.KO^ clone #1, only two of these genes (*ITGB2* and *COL12A1*) showed reduced expression in clone #2. Notably, *ITGB4*, *COL7A1*, *COL5A1* and *COL16A1* were selectively downregulated in A375^RIPK4.KO^ clone #1 but remained unchanged or were upregulated in clone #2. This divergence may underlie the distinct spheroid morphology and reduced cohesion observed specifically in clone #1. Gene expression patterns in WM266.4^RIPK4.KO^ cells showed greater alignment with those of A375^RIPK4.KO^ clone #1.

Additionally, both of these sublines (excluding A375^RIPK4.KO^ clone #2) exhibited reduced expression of *THBS1* and *CLEC3B*, along with elevated levels of *CD44*, *CTNND2* and *SPP1*.

Among the differentially expressed genes, several are known mediators of cell adhesion and matrix remodelling. *THBS1* encodes thrombospondin‐1, a trimeric glycoprotein involved in both cell–cell and cell–matrix interactions [31]. *CLEC3B*, which encodes tetranectin, participates in extracellular matrix remodelling through its C‐type lectin domain [32]. *CD44* is a ubiquitously expressed transmembrane receptor that regulates cell–matrix adhesion and exists in multiple isoforms [33]. *CTNND2* (delta‐catenin) plays a role in cell adhesion and motility [34], while *SPP1* (osteopontin) encodes a secreted matrix protein that enhances cell adhesion, migration and survival [35].

Despite differences between general genetic A375 and WM266.4 signatures, both lines exhibited overlapping signatures of ECM deregulation upon RIPK4 knockout. Notably, genes such as *COL1A1*, *COL4A2* and *SPP1* showed consistent upregulation, suggesting common downstream pathways impacted by RIPK4 deficiency. However, clone‐specific alterations, particularly in *ITGB4*, *COL7A1*, *THBS1* and *CTNND2* emphasize that the architectural impact of RIPK4 loss is tightly linked to the transcriptional context of individual subclones.

### 3.5 RIPK4 loss promotes incomplete pro‐amoeboid reprogramming in melanoma cells

**Fig. 5:**
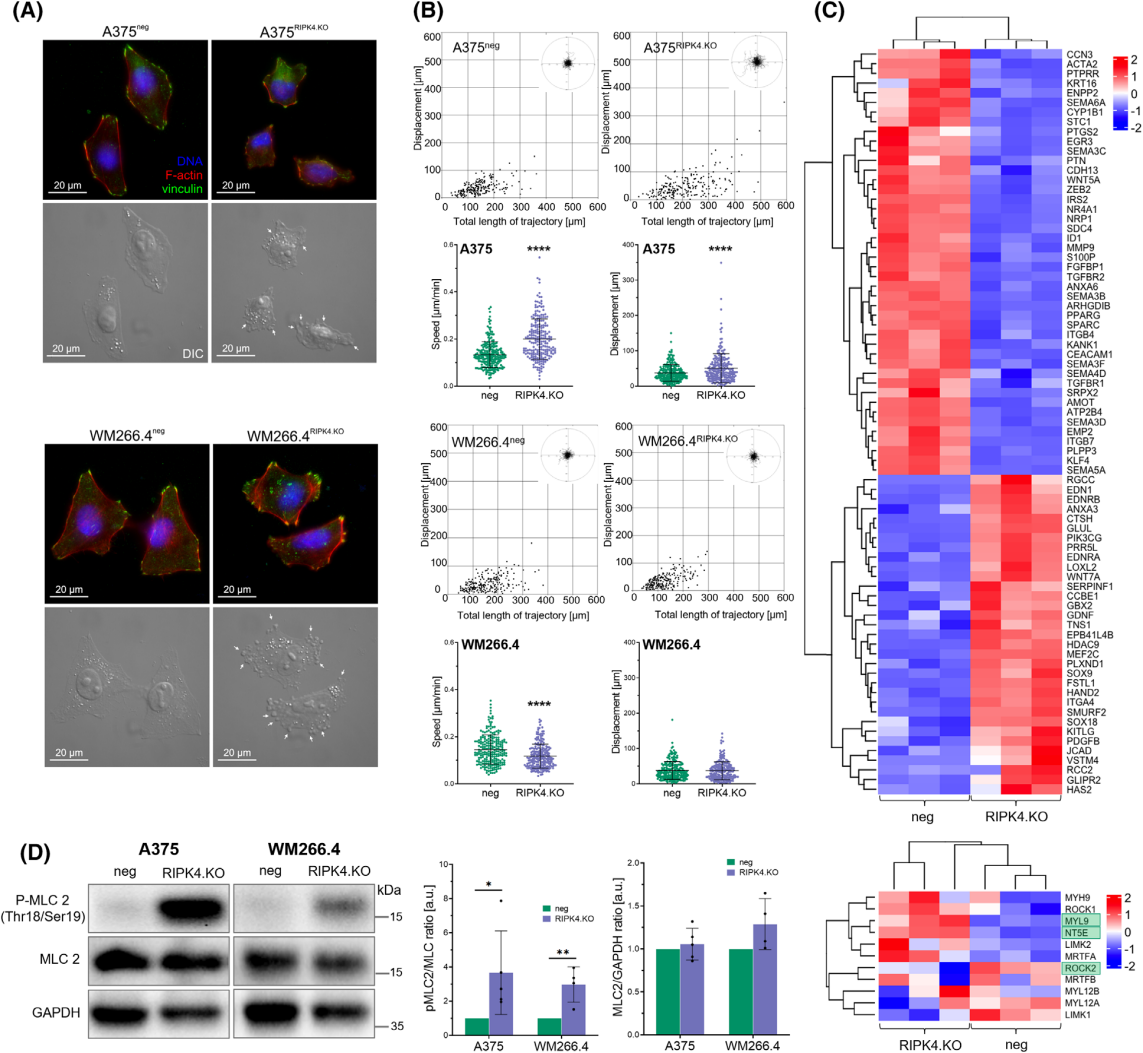
RIPK4 knockout promotes amoeboid phenotype of melanoma cells. (A) Morphology and cytoskeleton architecture of A375^RIPK4.KO^ (clone #1), WM266.4^RIPK4.KO^ and their respective negative controls. Vinculin (green) is visualized using TIRF microscopy; F‐actin cytoskeleton (red) and cell nuclei (blue) are visualized with epifluorescence microscopy. Corresponding differential interference contrast (DIC) images present cells' morphology. Bleb‐like structures are indicated with white arrows. Scale bars: 20 μm. (B) Motility of melanoma A375^RIPK4.KO^ (clone #1), WM266.4^RIPK4.KO^ and their respective negative control cells under 2D conditions. For each condition, data are presented as mean ± SD for *n* = 225 cells pooled from three independent experiments. Statistical analysis was performed using two‐tailed unpaired Student's *t*‐test. (C) RNA‐seq data from A375^RIPK4.KO^ (clone #1) and control cells, presented as heatmaps showing DEGs associated with the amoeboid mode of cell migration (upper panel; fold change > 1.5, FDR‐adj. *P* < 0.05) and the ROCK‐myosin II pathway (lower panel; genes highlighted in green indicate FDR‐adj. *P* < 0.05). Red‐blue gradient indicates the expression of transcript – red: upregulation; blue: downregulation. (D) Protein levels of phospho‐MLC2 and MLC2 detected by western blot. GAPDH was used as a loading control. A representative GAPDH band is shown. Bars represent mean ± SD from at least four independent biological replicates (*n* ≥ 4). Statistical analysis was performed using a two‐tailed unpaired Student's *t*‐test, *P < 0.05, **P < 0.01, ****P < 0.0001.

Prompted by the observed enrichment of amoeboid‐related gene signatures in RIPK4 knockout cells (Fig. 3), we examined their morphology and cytoskeletal dynamics in 2D culture. Cytoskeletal visualization of A375^RIPK4.KO^ (clone #1) and WM266.4^RIPK4.KO^ cells did not reveal any striking rearrangements of actomyosin in these cells in comparison to their respective control cells (Fig. 5A). Stress fibres and focal contacts were present in both populations, which is consistent with the parameters typically associated with the mesenchymal strategy of migration observed in our analysis (Fig. 5B). Notably, A375^RIPK4.KO^ (clone #1) cells displayed less flattened morphology, which was accompanied by slightly enhanced motility (Fig. 5B). In contrast, WM266.4^RIPK4.KO^ cells migrated more slowly. Despite these differences, both RIPK4.KO cell lines displayed signs of membrane blebbing, a morphological feature commonly associated with amoeboid cell migration (see arrows in Fig. 5A) [36, 37].

RNA‐seq analysis identified 77 differentially expressed genes (DEGs) in A375^RIPK4.KO^ (clone #1) cells, selected based on Gene Ontology (GO) enrichment analysis within the *Biological Process* category associated with amoeboid behaviour (Fig. 5C, Tables S1 and S2). These data revealed distinct transcriptomic shifts favouring amoeboid behaviour, characterized by downregulation of adhesion‐related genes and upregulation of cytoskeletal and contractility‐associated factors [38, 39]. Strong downregulation of genes encoding integrin subunits and adhesion molecules, such as *ITGB7*, *CEACAM1* and *CDH13*, suggests a widespread impairment of focal adhesion and cell–matrix interactions, which are critical for mesenchymal migration. Additionally, reduced expression of *MMP9* and *SPARC* may limit extracellular matrix remodelling, further promoting a switch to low‐adhesion motility modes. Conversely, downregulation or upregulation of several genes is known to enhance cortical contractility and promote blebbing. *ARHGDIB*, *PIK3CG*, *RGCC*, *WNT7A*, *SOX9* and *VSTM4* are associated with activation of Rho‐GTPase signalling, myosin II contractility and cytoskeletal plasticity. Among these, *PIK3CG* and *RGCC* are particularly implicated in actomyosin dynamics and have been previously linked to invasive bleb‐based motility. Upregulation of *PRR5L*, a component of the mTORC2 complex, may further contribute to membrane tension and bleb formation.

In our analysis, we also focussed on classical markers of RhoA‐ROCK‐myosin II driven blebbing, which are essential for actomyosin contractility and bleb‐based amoeboid migration. RIPK4 knockout led to significant upregulation of *MYL9*, known as *MLC2*, and *NT5E*, both key regulators of cortical tension and migration plasticity. *MYL9* encoding a myosin regulatory light chain 9 (also known as MLC2, MRLC1) enhances contractility upon ROCK‐mediated phosphorylation, supporting the observed shift toward bleb‐based motility. *ROCK2* expression was moderately downregulated (log_2_FC = −0.76; *P* = 0.0115), whereas *ROCK1* showed a mild but nonsignificant increase, suggesting a possible isoform‐specific compensation or signalling rebalancing within the ROCK pathway. Consistent with the transcriptomic changes, we observed a marked increase in phosphorylation of myosin light chain II (T18/S19), with an approximately fourfold increase (*P* < 0.05) in A375^RIPK4.KO^ cells and a nearly threefold increase (*P* < 0.01) in WM266.4^RIPK4.KO^ cells, indicating enhanced actomyosin activity (Fig. 5D).

Collectively, impaired cell flattening in RIPK4.KO cells, accompanied by deficient integrity of their spheroids, myosin II activation, enriched amoeboid phenotype‐associated transcriptomic signature, and the ‘mesenchymal’ morphology of RIPK4.KO cells on the rigid substratum suggests their ‘incomplete’ pro‐amoeboid reprogramming. In this context, the term ‘incomplete pro‐amoeboid reprogramming’ refers to a partial shift of RIPK4.KO cells towards amoeboid‐like characteristics observed at multiple levels: (a) transcriptionally, by upregulation of selected amoeboid‐associated genes without full acquisition of the canonical amoeboid transcriptomic profile; (b) morphologically, by increased cortical contractility, bleb formation, and partial cell rounding while retaining mesenchymal features on rigid substrates (i.e. pronounced focal adhesions); and (c) functionally, by reduced spheroid integrity and limited 3D motility compared to fully amoeboid cells. Together, these observations indicate a partial, rather than complete, shift toward the amoeboid phenotype. To our knowledge, this is the first report demonstrating that RIPK4 contributes to an incomplete amoeboid‐like reprogramming in melanoma cells, limiting their metastatic plasticity in 3D microenvironments.

### 3.6 RIPK4 knockout alters migration strategy and motility in 3D collagen matrices

To investigate whether amoeboid traits persist under more physiologically relevant conditions, we embedded RIPK4.KO cells in 3D collagen matrices and monitored their morphology and motility over time.

**Fig. 6:**
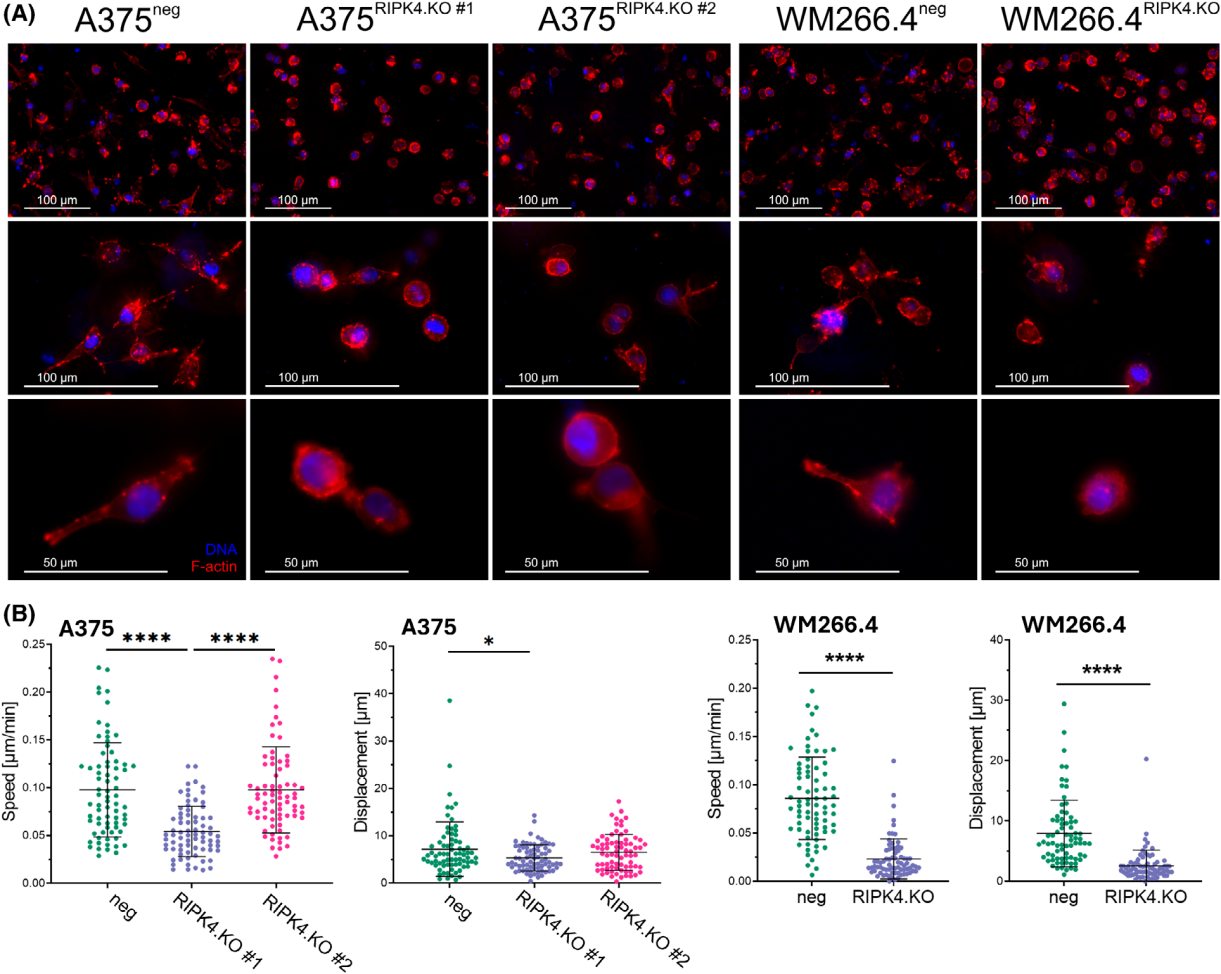
RIPK4 knockout promotes an incomplete amoeboid phenotype in collagen matrices. (A) Microphotographs of melanoma cells in 3D, presented as maximum projections of Z‐stacks captured at increasing magnifications and resolutions, showing the F‐actin cytoskeleton (red) and cell nuclei (blue). Top rows depict wider fields of view, while the bottom row shows higher magnifications of selected regions. Scale bar: 100 μm (top row and middle row) or 50 μm (bottom row). (B) Motile activity of melanoma A375^RIPK4.KO^ (clone #1 and #2) and WM266.4^RIPK4.KO^ cells and their negative controls in 3D during 3 h of time‐lapse recording. For each condition, motility was analysed in 75 individual cells, and data are presented as mean ± SD. Statistical analysis was performed using one‐way ANOVA or a two‐tailed unpaired Student's *t*‐test, *P < 0.05, ****P < 0.0001.

Amoeboid cells are typically characterized by a rounded, blebbing morphology and their movement does not require the activity of extracellular proteases. Instead, it depends on the intracellular hydrostatic pressure generated by actomyosin. Cells are also loosely adhered to the substratum and form protrusions based on cell cortex rupture rather than on intensive actin polymerization [40]. This phenomenon is usually more dominant in 3D conditions. More careful examination of A375^RIPK4.KO^ (clone #1) cells cultured in elastic collagen matrices (1.5 mg·mL^−1^) revealed the presence of scattered actin‐deficient blebs on their surfaces (Fig. 6A).

While A375^neg^ and WM266.4^neg^ cells rapidly developed actin‐rich, elongated protrusions typical for mesenchymal migration under these conditions (Fig. 6A, see Fig. S4a, Video S1), both A375^RIPK4.KO^ (clone #1) and WM266.4 cells behaved similarly, forming blebs in all directions (Fig. 6A, see Fig. S4a, Video S2). A375^RIPK4.KO^ (clone #1) and WM266.4^RIPK4.KO^ cells migrated less effectively than their control (neg) counterparts during the first 3 h of observation in 3D collagen matrices (Fig. 6B, Fig. S4b). This was reflected by reduced average migration speed and displacement, observed for both RIPK4.KO melanoma cells. However, between 3rd and 6th hour A375^RIPK4.KO^ began to form elongated, spike‐like protrusions comparable to negative cells and started to migrate in the ‘mesenchymal’ manner, confirming their incomplete pro‐amoeboid reprogramming (Videos S1 and S2). Additionally, A375^RIPK4.KO^ (clone #2) cells displayed an intermediate phenotype, exhibiting morphological and behavioural features between A375^RIPK4.KO^ clone #1 and their control cells (Fig. 6A), but with no significant impairment in migration (Fig. 6B). To further assess whether the initial morphology of RIPK4.KO cells reflects a contractility‐dependent amoeboid behaviour, we inhibited ROCK1/2 (Y‐27632, 10 μm) and myosin II (blebbistatin, 5 μm) during their migration in 3D collagen (Fig. S5a–c). ROCK inhibition caused only a minimal reduction in the migration speed of A375^RIPK4.KO^ (clone #1) cells, whereas blebbistatin led to a markedly stronger decrease in their motility (Fig. S5c). A similar trend was observed for WM266.4^RIPK4.KO^ cells, although the differences did not reach statistical significance. Notably, neither inhibitor completely abolished migration, which may be related to the compensatory formation of elongated, mesenchymal‐like protrusions observed under both conditions, appearing much earlier than in untreated cells (see Fig. S5a,b).

Together, these findings indicate that 3D culture conditions and reduced stiffness of the substratum transiently promote amoeboid behaviour of A375^RIPK4.KO^ (clone #1) and WM266.4^RIPK4.KO^ cells, which do not fully rescue the metastatic potential of melanoma cells.

### 3.7 RIPK4 rescue restore mesenchymal phenotype of A375^RIPK4.KO^ cells

**Fig. 7:**
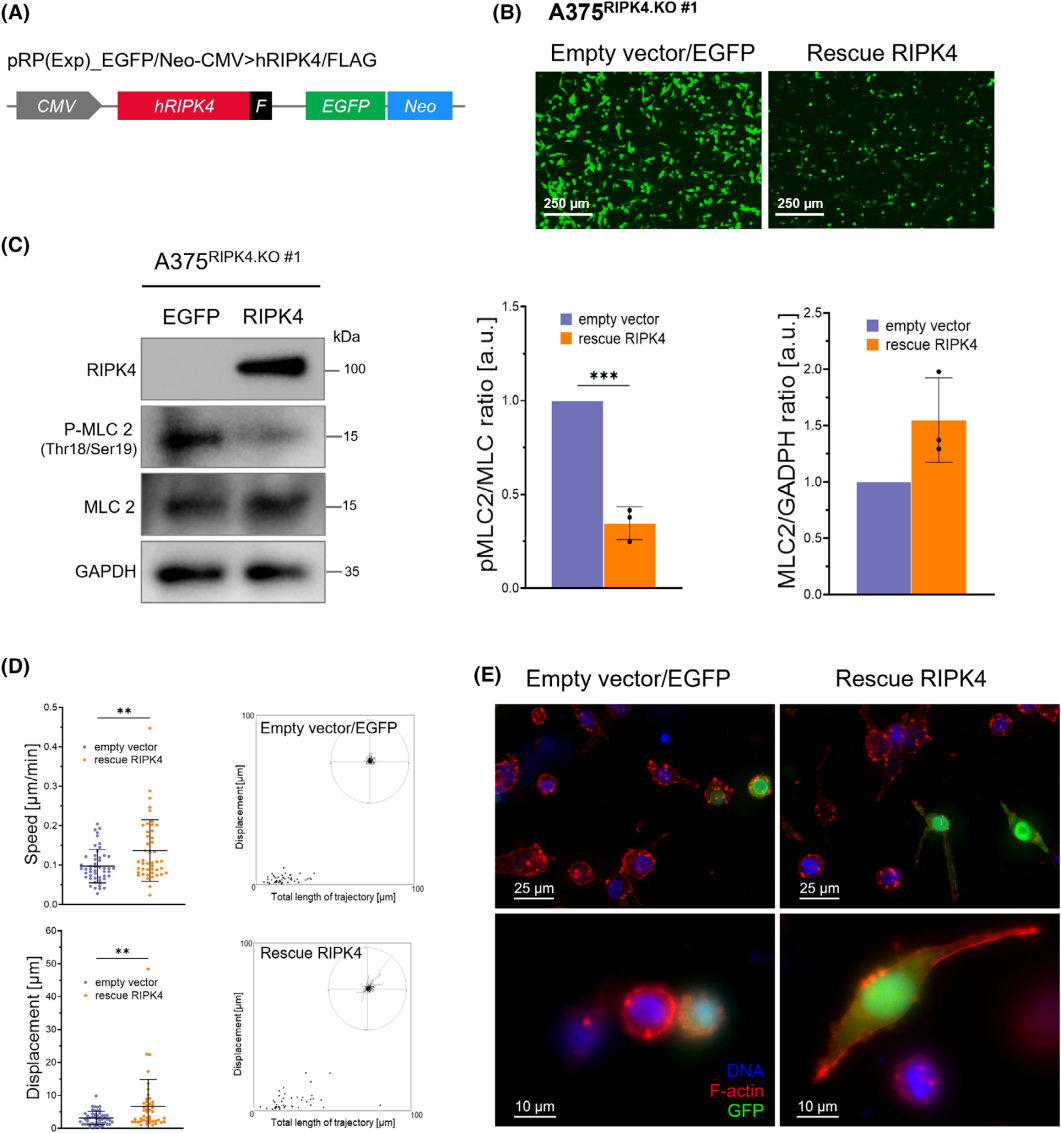
RIPK4 rescue restores a mesenchymal phenotype of A375^RIPK4.KO^ cells in collagen matrices. (A) A375^RIPK4.KO^ (clone #1) cells were transfected with a full‐length RIPK4 construct fused to a GFP tag, or with an empty GFP‐expressing vector as a control. (B) Representative image of GFP following transfection. Scale bar = 250 μm. (C) Western blot analysis of RIPK4, phospho‐MLC2 and total MLC2 levels. GAPDH was used as a loading control. Densitometric quantification is presented as bar plots showing the mean ± SD from three independent biological replicates (*n* = 3). (D) Quantification of migration of individual GFP‐positive cells in 3D collagen gel over 3 h of time‐lapse imaging. Data are presented as mean ± SD, each dot represents a single cell from *n* = 50 cells (empty vector/EGFP) or *n* = 45 cells (RIPK4 rescue). (E) Microphotographs of melanoma cells in 3D, presented as maximum projections of Z‐stacks (upper row) or as magnified single‐plane images (bottom row), showing the F‐actin cytoskeleton (red), GFP (green) and cell nuclei (blue). Scale bar: 25 μm (upper row) and 10 μm (bottom row). Statistical analysis was performed using two‐tailed unpaired Student's *t*‐test, **P < 0.01, ***P < 0.001.

To assess whether re‐expression of RIPK4 could reverse the migratory phenotype associated with MLC2 activity following RIPK4 loss, we introduced a RIPK4‐expressing plasmid (Fig. 7A) into A375^RIPK4.KO^ cells (clone #1). Cells transfected with an empty vector served as controls. Successful transfection was confirmed by GFP fluorescence, indicating plasmid uptake (Fig. 7B, see Fig. S6), and by western blotting for the FLAG‐tagged RIPK4 protein (Fig. 7C). A375^RIPK4 rescue^ cells showed restored RIPK4 protein levels and a significant reduction of MLC2 phosphorylation compared to empty vector control cells (Fig. 7C). Live‐cell tracking in 3D collagen gels demonstrated that the A375^RIPK4 rescue^ cell population displayed increased motility relative to controls (Fig. 7D). Morphologically, a subset of these cells adopted an elongated, spindle‐like shape characteristic of mesenchymal migration, an appearance absent in the empty vector group (Fig. 7E). These findings indicate a functional link between RIPK4 and MLC2‐driven contractility, which may contribute to alterations in melanoma cell migration dynamics.

## 4 Discussion

Melanoma's aggressiveness and treatment resistance stem from its high metastatic plasticity and ability to switch between migration modes in response to microenvironmental cues [41]. Intriguingly, the studies indicate that RIPK4 can regulate epithelial‐to‐mesenchymal transition (EMT) through the STAT3 pathway, although the functional consequences appear to be highly context‐dependent. In hepatocellular carcinoma, RIPK4 suppresses EMT and metastasis by inhibiting STAT3 phosphorylation, whereas in ovarian cancer, it enhances EMT through IL‐6/STAT3 signalling, thereby promoting invasiveness [42, 43]. Our findings reveal that RIPK4 supports this invasive adaptability, and its loss triggers a shift toward an amoeboid‐like state with cellular and environmental context‐specific effects on the metastatic potential of melanoma cells. They are consistent with the oncogenic function of RIPK4 in melanoma [14, 15]. In melanoma, which originates from neural crest‐derived melanocytes, RIPK4 may be hijacked to support invasive flexibility and phenotypic switching, both essential traits for efficient metastasis. Our current observations also expand the context‐dependent understanding of RIPK4's involvement in melanoma progression, particularly by delineating its function in advanced phases of disease spread, where dynamic interactions with the microenvironment become critical. Specifically, they confirm: (a) the crucial role of RIPK4 in driving melanoma cell invasiveness and the metastatic cascade; executed via (b) coordination of regulatory systems involved in the maintenance of cell adhesion. They also show extensive transcriptomic reprogramming, along with the emergence of an amoeboid‐like phenotype in RIPK4‐deficient melanoma cells, which indicates (c) the activation of compensatory mechanisms that partially mitigate RIPK4 dysfunction.

Using a combination of clinical samples and functional assays, we show that RIPK4 expression increases with melanoma progression, peaking in metastatic lesions. This is consistent with our *in vivo* tail‐vein model data, where RIPK4‐deficient cells formed fewer and smaller lung lesions, suggesting that RIPK4 primarily supports late metastatic colonization rather than the earlier steps of dissemination. Mechanistically, transcriptomic profiling has revealed that RIPK4 is involved in regulating the expression of genes related to extracellular matrix organization and adhesion molecule signalling, two key determinants of invasion. These findings are consistent with our prior siRNA‐based studies that linked RIPK4 to EMT‐regulating pathways, including Wnt/β‐catenin and NF‐κB signalling [14, 15]. Amoeboid migration, characterized by high actomyosin contractility and cell rounding, plays a crucial role in melanoma metastasis and is regulated by numerous cytokines, including TGF‐β. Interestingly, while TGF‐β typically induces EMT in many cancers, in melanoma, it paradoxically promotes amoeboid features, likely due to the neural crest origin of melanocytes [44]. Our RNA‐seq analysis revealed that RIPK4 silencing leads to reduced expression of TGFBR1 and TGFBR2, suggesting that RIPK4 positively modulates TGF‐β signalling. This aligns with previous findings showing that, in melanoma, RIPK4 functions as an oncogene [14, 45] that regulates key signalling pathways such as NF‐κB, Wnt and MAPK, all crucial for melanoma plasticity and migration [14]. Notably, we observed downregulation of WNT5A in RIPK4.KO cells, which has been shown to suppress amoeboid migration when overexpressed [46]. As RIPK4 is known to promote Wnt signalling [15, 47, 48, 49], its loss may reduce WNT5A‐driven mesenchymal motility, thereby contributing to the amoeboid phenotype.

Cancer cell extravasation and colonization of distant organs critically depend on the adhesive interaction between circulating tumour cells and the endothelium [50]. Key adhesion receptors such as CD146 (originally identified as a melanoma cell adhesion molecule—MCAM), JAM‐C and EphA2 facilitate this process by promoting endothelial binding and transendothelial migration [28, 51]. We show that RIPK4 knockout led to reduced expression of adhesion molecules, for example N‐cadherin and MCAM, and variable effects on JAM‐C. Interestingly, while changes in the expression of these adhesion molecules were consistently observed, the magnitude of the effect varied between clones and cell lines. Previously, Ghislin et al. [52] demonstrated that JAM‐C expression differs between melanoma lines, being higher in A375 than in SLM8, and that JAM‐A regulates JAM‐C surface localization, with a stronger effect in A375. Similarly, recent findings by Mannion et al. have shown that CD146/MCAM displays heterogeneous expression across breast cancer subtypes, with its function shaped by epithelial‐to‐mesenchymal plasticity, emphasizing the context‐dependent behaviour of this adhesion molecule. The variability observed in our study likely reflects the well‐documented intra‐ and intertumoral heterogeneity and subclonal genetic and phenotypic diversity of melanoma [53, 54]. It is further amplified by the high degree of plasticity in melanoma cells, which allows dynamic switching between invasive and proliferative states in response to environmental stimuli [55]. This plasticity is a key driver of melanoma progression and therapeutic resistance, highlighting the importance of accounting for clonal diversity in mechanistic studies and when designing targeted interventions.

Context dependence of RIPK4 function is further evident when comparing our stable RIPK4 knockout models with previous siRNA‐based experiments in the same cell lines, where no change in N‐cadherin expression was observed, likely due to the transient and incomplete nature of siRNA knockdown [14]. This distinction underscores the importance of stable gene editing in uncovering the long‐term phenotypic consequences. Our current data demonstrate that loss of RIPK4 impairs the invasive capacity of melanoma cells. These findings suggest that RIPK4 not only integrates pro‐invasive signalling pathways but also maintains phenotypic stability of melanoma cells. Apparently, phenotypic shifts between epithelioid, mesenchymal and amoeboid morphology govern the invasive potential of melanoma cells via RIPK4‐responsive signalling pathways.

Basic morphological traits of cancer cells are related to the distinct strategies (collective, mesenchymal and amoeboid) of cell movement [56, 57]. Incomplete mesenchymal‐amoeboid transition is highly relevant for cancer development, particularly in promoting metastasis and tumour invasiveness. It can result in a ‘hybrid’ or ‘plastic’ phenotype, which enables tumour cells to dynamically switch between different modes of movement based on the extracellular environment [41, 58, 59, 60]. Such hybrid mesenchymal‐amoeboid cells can adjust their shapes and motility to overcome physical barriers. Mesenchymal migration enables cells to remodel the extracellular matrix, while amoeboid movement allows for squeezing through small spaces, thus enhancing cellular invasiveness. Incomplete mesenchymal‐amoeboid transition, similar to incomplete epithelial–mesenchymal transition, may also be associated with enhanced metabolic plasticity and increased drug resistance [61]. Cells may thus more effectively interact with stromal cells within the tumour microenvironment. Together, these traits facilitate both initial tumour dissemination and invasion into secondary sites [62].

**Fig. 8:**
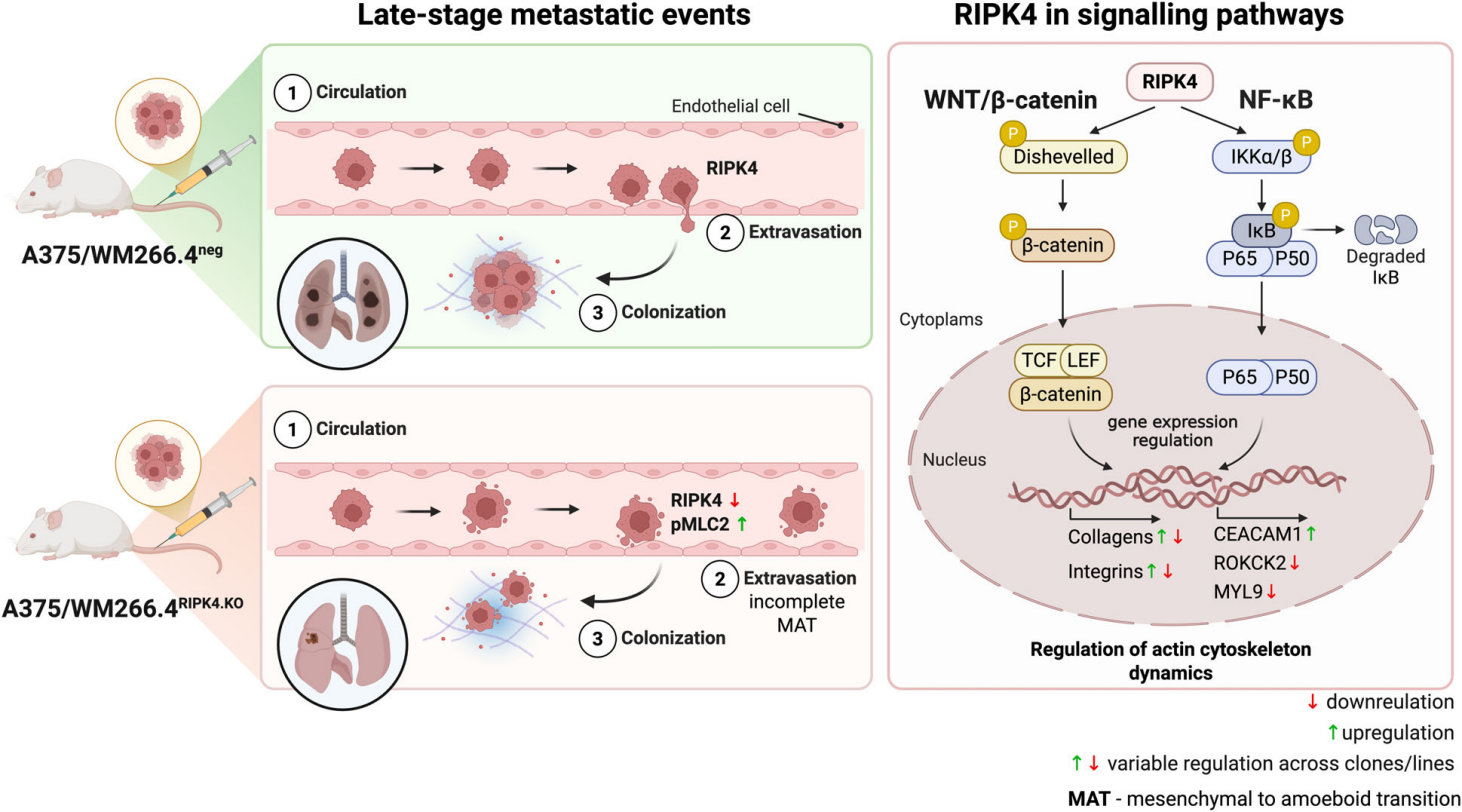
Schematic summary illustrating the role of RIPK4 in late‐stage metastatic events and intracellular signalling. The left panel depicts late‐stage metastatic events *in vivo*: circulating melanoma cells with normal RIPK4 levels efficiently extravasate and colonize lung tissue, whereas RIPK4‐deficient cells exhibit ‘incomplete’ pro‐amoeboid reprogramming, associated with altered pMLC2 signalling, resulting in reduced extravasation, impaired migration and diminished lung colonization. The right panel illustrates how RIPK4 integrates WNT/β‐catenin and NF‐κB signalling pathways to regulate downstream transcriptional programmes involving collagens, integrins, CEACAM1, MYL9 and ROCK2, thereby modulating actomyosin cytoskeleton dynamics. Created in BioRender. Madej, E. (2025) https://BioRender.com/9npa7he.

Our data reveal that RIPK4 knockout induces features of amoeboid migration, including membrane blebbing and increased MLC2 phosphorylation, yet does not fully support efficient amoeboid motility. RNA‐seq and protein level analysis revealed a partial upregulation of amoeboid effectors such as MYL9, RGCC and PIK3CG, but also a downregulation of essential components such as ROCK2 and adhesion inhibitors (e.g. CEACAM1), indicative of an incomplete pro‐amoeboid reprogramming. While the precise positioning of RIPK4 within this regulatory network remains to be fully defined, our data indicate that its loss alters the expression of key cytoskeleton‐related proteins, including ROCK2 and CEACAM1, as well as MYL9. Notably, CEACAM1 is transcriptionally regulated by the NF‐κB subunit p65 [63], while MYL9 is a Wnt/β‐catenin‐dependent target gene [64], both pathways in which RIPK4 exhibits activity in melanoma [14, 15, 65]. This suggests that RIPK4 does not regulate cytoskeletal dynamics directly but rather acts upstream by modulating signalling cascades that control the transcription of cytoskeletal effectors. This is illustrated in the schematic (Fig. 8).

An important aspect of this control is a disturbed balance between ROCK isoforms in RIPK4.KO cells. While individual heatmaps indicated an increase in ROCK1 expression, ROCK2 levels were markedly reduced. A growing body of evidence shows that the relative abundance of ROCK isoforms can strongly influence migratory behaviour across diverse microenvironments. Both overlapping and distinct functions of ROCK1 and ROCK2 have been comprehensively reviewed by Wei et al. [66]. Although the authors emphasize that isoform‐specific contributions are highly cell‐type dependent, most studies cited therein highlight the requirement of both isoforms for efficient migration, albeit through their effects on different aspects of cytoskeletal dynamics. More recent reports support this view. In MDA‐MB‐231 breast cancer cells migrating on substrates of varying stiffness, ROCK1 primarily governed actomyosin contractility through MLC phosphorylation, whereas ROCK2 controlled actin polymerization within directionally stabilized protrusions [67]. Consistently, depletion of ROCK1 reduced cell spreading and focal adhesion size [68], while ROCK2 loss impaired invadopodia formation and diminished transwell migration, indicating a reduced mesenchymal invasive capacity [69]. Collectively, these findings support the notion that alterations in the ROCK1/ROCK2 expression ratio can shift the balance between amoeboid‐ and mesenchymal‐like behaviours, promoting intermediate or hybrid migratory phenotypes.

Importantly, live‐cell tracking in compliant collagen matrices confirmed that RIPK4‐deficient melanoma cells initially adopted a rounded, blebbing phenotype but failed to sustain efficient movement. Over time, these cells partially reverted to a mesenchymal‐like morphology, which was further accelerated upon pharmacological inhibition of myosin II activity. Such dynamic plasticity resembles the ‘intermediate’ migratory states described in recent single‐cell profiling of melanoma invasion [70] and supports the hypothesis that RIPK4 acts as a molecular integrator of the migration strategy. In our hands, impaired invasiveness of RIPK4.KO cells was accompanied by the signs of their pro‐amoeboid reprogramming. Considering the generally enhanced invasiveness of amoeboid cells [41, 58, 59, 60] and the pro‐invasive role of RIPK4 in melanoma systems [14], this observation was somewhat unexpected. It can, however, be interpreted in terms of the incomplete character of pro‐amoeboid reprogramming in RIPK4.KO melanoma cells. Upon embedding in collagen, these cells transiently adopt a rounded, blebbing morphology. Despite elevated MLC2 phosphorylation, they fail to generate effective forward movement during this phase and subsequently transition toward elongated, protrusive shapes, partially restoring motility. This behaviour confirms that RIPK4.KO cells do not achieve a fully functional amoeboid state but instead enter a short‐lived, mechanically ineffective amoeboid‐like configuration, consistent with incomplete amoeboid reprogramming.

Notably, the weak response of RIPK4.KO cells to ROCK inhibition—despite increased myosin II activity—suggests that contractility may be regulated through pathways other than canonical ROCK‐MLC2 signalling. Recent work demonstrated that AMPK can enhance myosin II‐dependent tension by phosphorylating MYPT1 at Ser472, thereby inhibiting myosin phosphatase and promoting contractility in 3D environments [71]. Although we did not directly test this mechanism, the observation that blebbistatin—but not ROCK inhibition—reduces motility of RIPK4.KO cells raises the possibility that alternative regulators of myosin II contribute to the incomplete and non‐productive amoeboid‐like state observed in our system. Although this phenotype impairs invasiveness, it represents an intermediate stage towards acquisition of a fully amoeboid phenotype, as described for A375 cells [40, 72]. This interpretation could also explain why some RIPK4‐negative melanoma cell lines retain relatively high invasiveness. An open question remains whether pro‐amoeboid reprogramming of melanoma cells results from extensive rearrangement of their secretome (especially of ECM‐related proteins) or both processes are parallels, determined by other factors. For instance, RIPK4‐related epigenetic cues, like mechanical equilibrium of cytoskeleton and nuclear architecture [73, 74], can facilitate compensatory shifts in cancer cells and their reprogramming.

Collectively, our results demonstrate that RIPK4 supports melanoma cell invasiveness by maintaining the transcriptional and cytoskeletal coherence necessary for efficient mesenchymal–amoeboid plasticity. Its loss triggers an incomplete pro‐amoeboid reprogramming that compromises the migration efficiency of melanoma cells but may initiate adaptive responses leading to partial recovery of their invasiveness. Consequently, RIPK4 primarily modulates the early stages of the metastatic cascade, including the tumour cell invasion and the adhesion‐dependent intravasation/extravasation via the effect on the expression of genes related to extracellular matrix organization and adhesion. Importantly, *in vivo* tail vein injection experiments demonstrated markedly fewer and smaller lung metastatic foci formed by RIPK4‐deficient cells, indicating their impaired ability to efficiently extravasate and/or colonize secondary sites (Fig. 8). These findings also suggest that RIPK4 contributes to later stages of metastatic colonization. However, since the tail vein model bypasses the intravasation step, these results cannot definitively separate the contribution of RIPK4 to extravasation, survival of circulating tumour cells or metastatic outgrowth aspects that warrant further targeted investigation.

Nevertheless, our observations demonstrate a novel, previously unaddressed aspect of the RIPK4 function, which points to its role in key cellular processes related to migration and adhesion. While our study was conducted using two independent melanoma cell lines, both exhibiting variable degrees of RIPK4 dependence, these findings may not fully represent the entire spectrum of melanoma subtypes. Therefore, these data should be interpreted as indicative of a context‐dependent rather than universal role of RIPK4 in melanoma metastasis. However, our insights extend the current understanding of how melanoma cells balance adhesive and contractile forces during metastatic progression and highlight RIPK4 as a potential target for therapeutic modulation of tumour cell plasticity.

## 5 Conclusions

Our study demonstrates that RIPK4 is a key regulator of melanoma cell invasiveness, maintaining transcriptional and cytoskeletal coherence required for mesenchymal–amoeboid plasticity. Loss of RIPK4 induces incomplete pro‐amoeboid reprogramming, with transient blebbing and altered expression of adhesion and cytoskeletal effectors, which limits migration but triggers compensatory mechanisms. RIPK4 coordinates key signalling pathways, including TGF‐β, Wnt and NF‐κB, regulating extracellular matrix organization, adhesion molecule expression and cytoskeletal dynamics. *In vivo*, RIPK4‐deficient cells form fewer and smaller lung metastases, indicating impaired colonization and reduced late‐stage metastatic potential. These findings highlight RIPK4 as a context‐dependent oncogene that integrates adhesive and contractile signals to support melanoma progression and represents a potential therapeutic target.

## Conflict of interest

The authors declare no conflict of interest.

## Author contributions

NW was involved in investigation, visualization, formal analysis, review and editing. SL, EM, AAB were involved in investigation, visualization, formal analysis, and writing—manuscript review and editing. MS was involved in investigation. AH‐L was involved in investigation and formal analysis. JC was involved in investigation. JR was involved in formal analysis. J Czyz was involved in writing of the original draft and writing—manuscript review and editing. AW‐G was involved in conceptualization and study design, writing of the original draft, supervision, project administration, funding acquisition and writing—manuscript review and editing.

## Ethics approval and consent to participate

The clinical study was conducted in accordance with the Declaration of Helsinki (1975, revised in 2008) and approved by the Institutional Review Board of the Collegium Medicum, Nicolaus Copernicus University (KB136/2016), and by the Bioethics Committee of the Jagiellonian University (1072.6120.125.2017). Written informed consent was obtained from all participants. Animal experiments were approved by the II Local Ethics Committee of the Institute of Pharmacology of the Polish Academy of Sciences (approval numbers: 82/2023, 135/2023, and 52/2024).

## Supporting information


**Fig. S1.** Chromatograms showing sequence analysis to confirm the mutation in the RIPK4 target region in A375 clone #2.
**Fig. S2.** RNA‐seq analysis revealed significant changes in gene expression after RIPK4 knockout in A375 cells.
**Fig. S3.** Validation of selected DEGs.
**Fig. S4.** Motility and behaviour of melanoma cells in 3D environments.
**Fig. S5.** Effect of ROCK and myosin II inhibition on melanoma cell motility in 3D collagen matrices.
**Fig. S6.** Representative GFP fluorescence and bright‐field images of A375^RIPK4.KO^ cells transfected with either an empty vector/EGFP or a rescue RIPK4 construct.

**Table S1.** List of upregulated DEGs related to amoeboidal behaviour.

**Table S2.** List of downregulated DEGs related to amoeboidal behaviour.

**Video S1.** A375^negative^ cells migration in a 3D collagen matrix.

**Video S2.** A375^RIPK4.KO clone #1^ cells migration in a 3D collagen matrix.

## Data Availability

Data are available at https://uj.rodbuk.pl. RNA‐seq data have been deposited in GEO under accession number GSE314542. Other data supporting the findings of this study are available from the corresponding author upon reasonable request.
