# Supplementary material for: RIPK4 function interferes with melanoma cell adhesion and metastasis
Source: Mol Oncol. 2026 Feb 9;20(5):1115–39. doi: 10.1002/1878-0261.70220 (PMC13155153; doi:10.1002/1878-0261.70220)
Supplement: Supplementary file 1 — Fig. S1. Chromatograms showing sequence analysis to confirm the mutation in the RIPK4 target region in A375 clone #2. Fig. S2. RNA‐seq analysis revealed significant changes in gene expression after RIPK4 knockout in A375 cells. Fig. S3. Validation of selected DEGs. Fig. S4. Motility and behaviour of melanoma cells in 3D environments. Fig. S5. Effect of ROCK and myosin II inhibition on melanoma cell motility in 3D collagen matrices. Fig. S6. Representative GFP fluorescence and bright‐field images of A375RIPK4.KO cells transfected with either an empty vector/EGFP or a rescue RIPK4 construct. [file MOL2-20-1115-s003.docx]

*Supplementary Materials*

**RIPK4 function interferes with melanoma cell adhesion and metastasis**

Norbert Wronski^1,2^, Sławomir Lasota^3^, Ewelina Madej^1^, Anna A. Brożyna^4^, Małgorzata Szczygieł^1^, Agnieszka Harazin-Lechowska^5^, Jan Czerbniak^1^, Janusz Rys^5^, Jaroslaw Czyz^3^, Agnieszka Wolnicka-Glubisz^1*^

^1^ Department of Biophysics and Cancer Biology, Faculty of Biochemistry, Biophysics and Biotechnology, Jagiellonian University, Gronostajowa 7 street, Krakow, Poland

^2^ Doctoral School of Exact and Natural Sciences, Jagiellonian University, Krakow, Poland

^3^ Department of Cell Biology, Faculty of Biochemistry, Biophysics and Biotechnology, Jagiellonian University, Gronostajowa 7 street, Krakow, Poland

^4^ Department of Human Biology, Institute of Biology, Faculty of Biological and Veterinary Sciences, Nicolaus Copernicus University, Lwowska 1 street, Torun, Poland

^5^ Department of Tumour Pathology, Maria Sklodowska-Curie National Research Institute of Oncology, Krakow Branch, Garncarska 11 street, Krakow, Poland

Correspondence: A. Wolnicka-Glubisz, Department of Biophysics and Cancer Biology, Faculty of Biochemistry, Biophysics and Biotechnology, Jagiellonian University, Gronostajowa Street 7, 30-387 Kraków, Poland, tel. +48 12 664 65 26, email: [a.wolnicka-glubisz@uj.edu.pl](mailto:a.wolnicka-glubisz@uj.edu.pl)


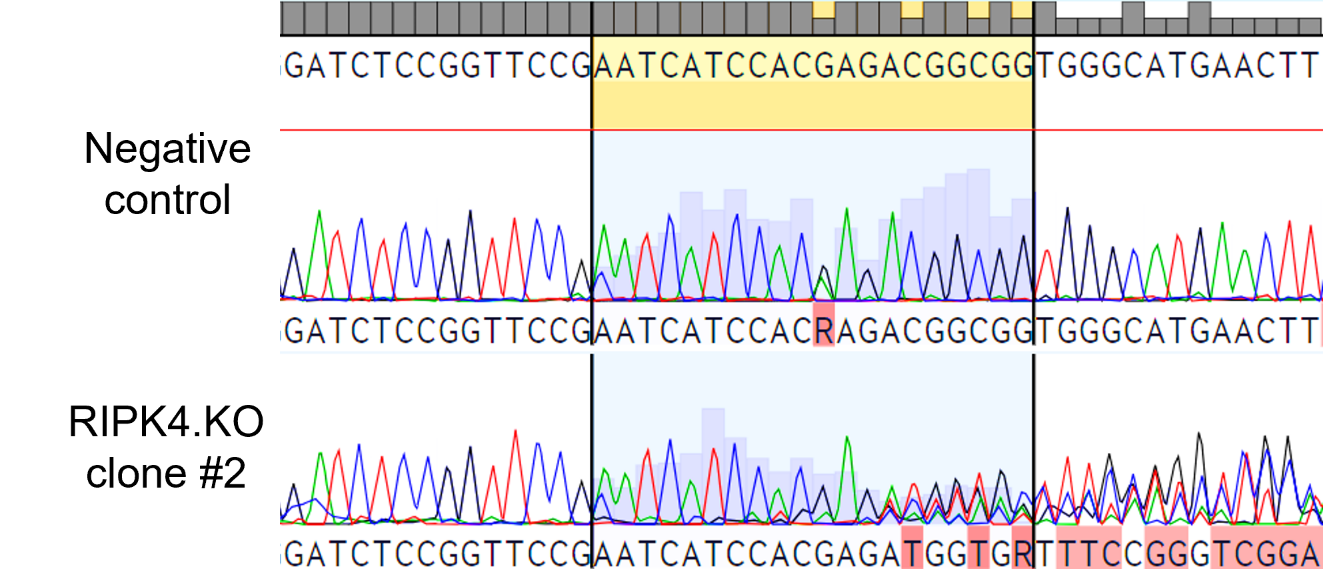


**Fig. S1.** **Chromatograms showing sequence analysis to confirm the mutation in the RIPK4 target region in A375 clone #2.**


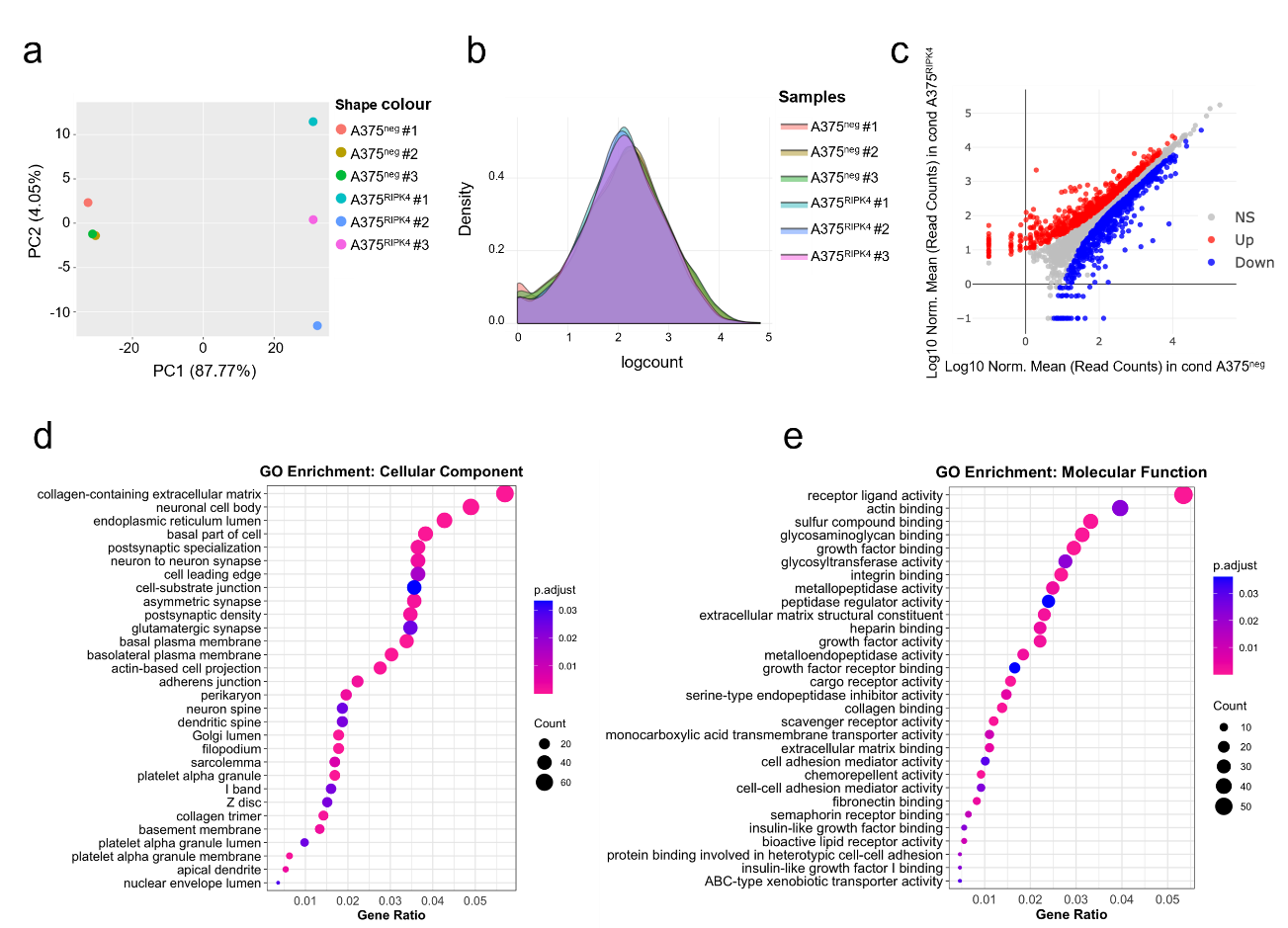
.

**Fig. S2. RNA-seq analysis revealed significant changes in gene expression after RIPK4 knockout in A375 cells.** (**a**) Principal component analysis of RNA-seq samples. Each dot represents one biological replicate. Samples from RIPK4 knockout (A375^RIPK4.KO^ clone #1) and control (A375^neg^ ) cells form distinct clusters, indicating clear transcriptomic separation. (**b**) Density plot showing the distribution of log-transformed gene expression values across all samples. All replicates exhibit comparable global expression profiles, confirming effective normalization and similar transcriptomic complexity. (**c**) A scatter plot displaying differential gene expression between A375^RIPK4.KO^ and control cells. Red and blue dots represent significantly upregulated and downregulated genes, respectively (FDR < 0.05; |log₂FC| > 1.5), while grey dots indicate non-significant changes. (**d, e**) GO enrichment analysis of DEGs identified by RNA-seq. The bubble plot displays the top 30 significantly enriched (**d)** Cellular Component and (**e**) Molecular Function. The size of each bubble represents the number of DEGs associated with each term, and the colour indicates statistical significance. Statistical analysis was performed using DESeq2 based on RNA-seq data from three independent biological replicates per condition. (n=3).

*
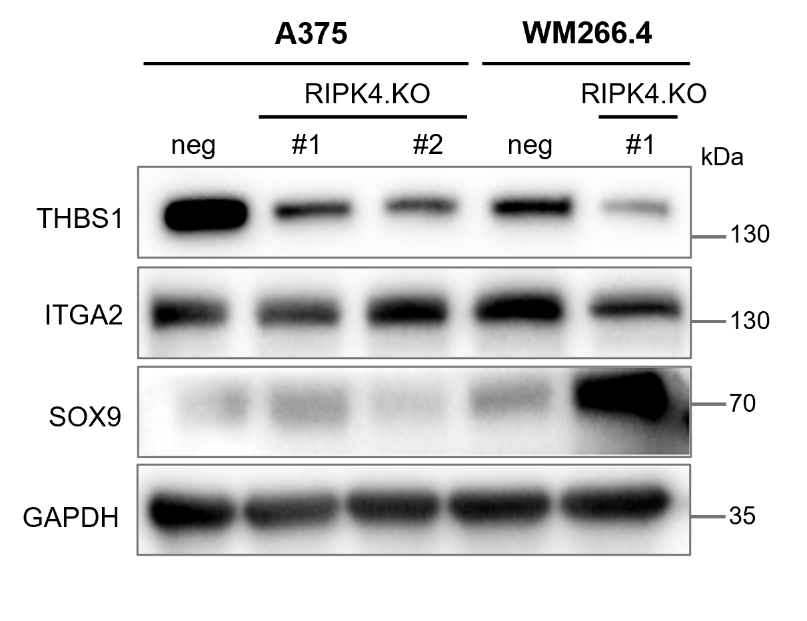
*

**Fig. S3. Validation of selected DEGs at the protein level.** Protein levels of SOX9, ITGA2, and THBS1 were assessed by Western blotting analysis (n = 1) to confirm RNA-seq–identified transcriptional changes.


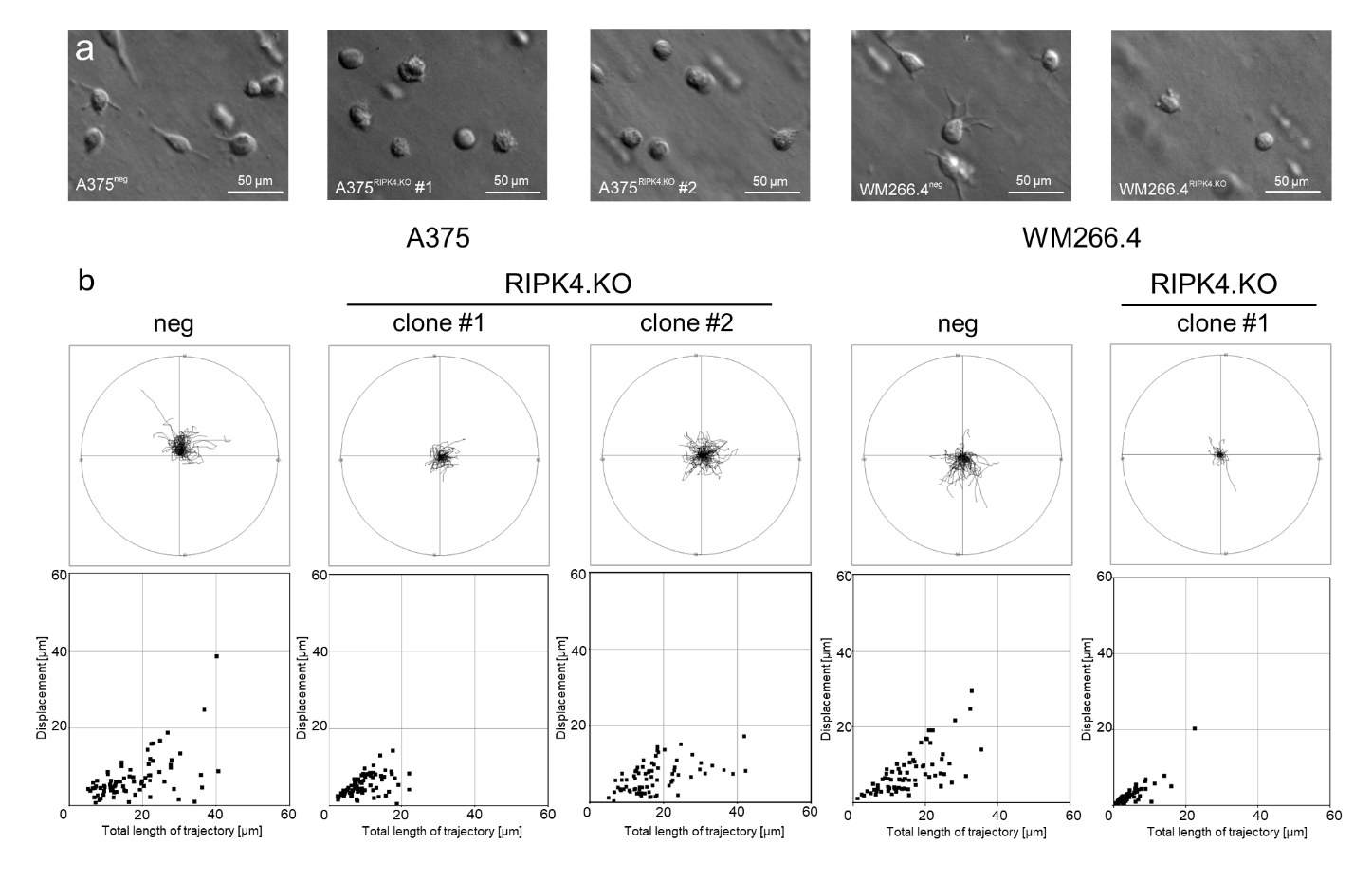


**Fig. S4. Motility and behavior of melanoma cells in 3D environments.** (**a**) Microphotographs of melanoma cells in 3D conditions captured with IMC. Scale bar: 50 µm. (**b**) The motile activity of melanoma A375 ^RIPK4.KO^ (clone #1 and #2) and WM266.4^RIPK4.KO^ cells and their negative control in 3D during 3 hours of time lapse imaging, presented as dot plots and circular diagrams ( each dot represents an individual cell). For each condition, motility was analyzed in 75 individual cells.


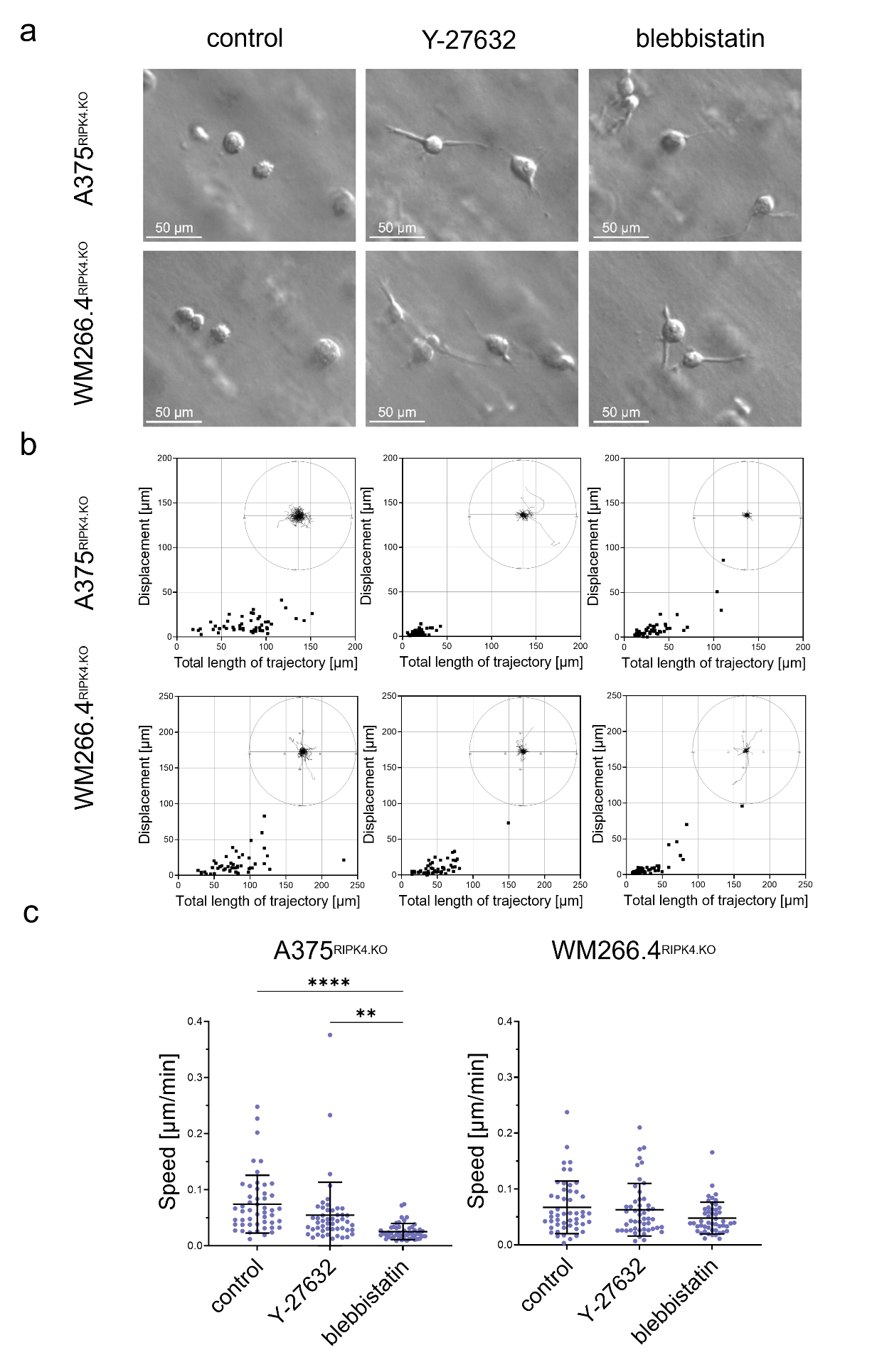


**Fig. S5.** **Effect of ROCK and myosin II inhibition on melanoma cell motility in 3D collagen matrices. (a)**Microphotographs of A375^RIPK4.KO^ (clone #1) and WM266.4^RIPK4.KO^ melanoma cells after 3 hours under control conditions in 3D collagen matrices or following treatment with Y-27632 (10µM) or blebbistatin (5µM), acquired using IMC. Scale bar: 50 µm. (**b**) Motile activity of cells incubated with the inhibitors indicated above during 3 hours of time-lapse imaging, presented as dot plots and circular diagrams. **(c)** The motility of cells presented quantitatively as mean ± SD for n = 50 cells per condition, derived from a single biological experiment. Statistical analysis was performed using one-way ANOVA ** p < 0.01, **** p < 0.0001.


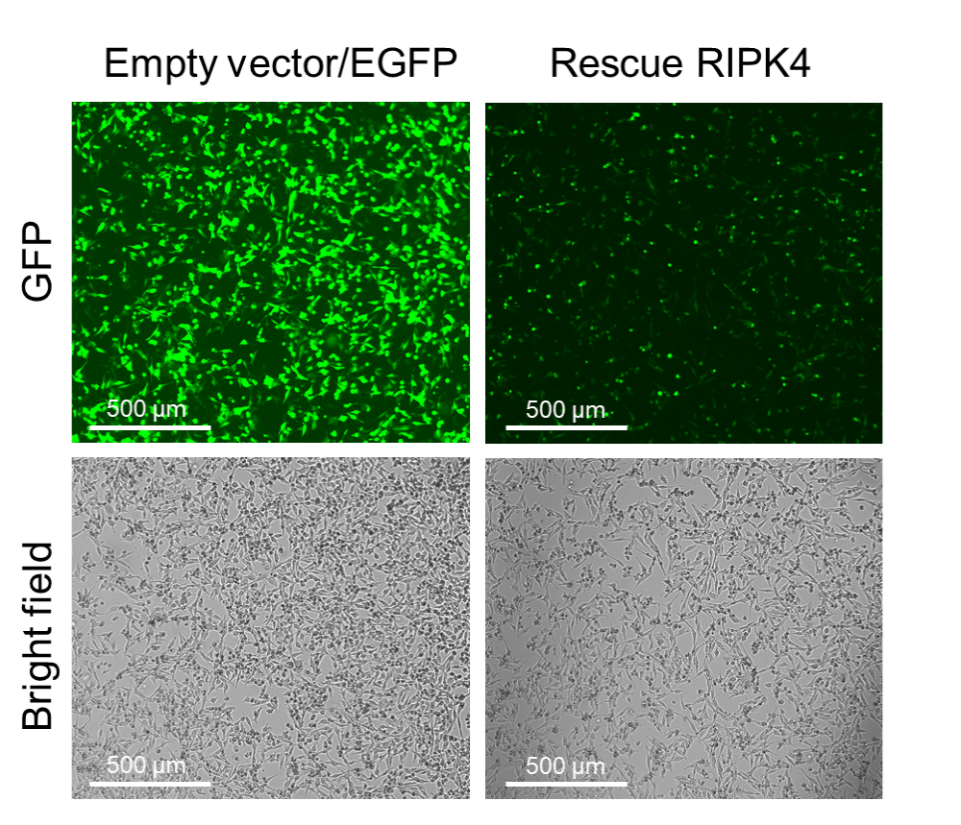


**Fig. S6.** **Representative GFP fluorescence and bright-field images of A375^RIPK4.KO^ cells transfected with either an empty vector/EGFP or a rescue RIPK4 construct.** Scale bar: 500 µm.

**Supplementary Videos Captions**

Videos related to supplementary figure S2 present A375 cell migration in a 3D collagen matrix (1.5 mg/ml). Both videos were recorded over 6 hours with a 5-minute time interval using IMC. One representative slice from the Z-stack is shown. Scale bar = 50 μm. Frame rate: 10 fps. Compression MPEG-4 in mp4 container.

**Supplementary Video S1** A375^negative^ cells migration in a 3D collagen matrix.

**Supplementary Video S2** A375^RIPK4.KO clone #1^ cells migration in a 3D collagen matrix.
